# A multiplex serological assay for quantification of arbovirus antibody kinetics and neutralizing antibodies

**DOI:** 10.1371/journal.pntd.0013704

**Published:** 2026-09-23

**Authors:** Laura Garcia, Victor Yman, Marie-Fabrice Gasasira, Françoise Donnadieu, Jason Rosado, Lucie Cappuccio, Damien Hoinard, Philippe Desprès, Stéphane Petres, Jean-Claude Manuguerra, In-Kyu Yoon, Taweewan Hunsawong, Maria Theresa P. Alera, Emily S. Gurley, Nikos Vasilakis, Scott C. Weaver, Ziaur Rahman, Ilaria Dorigatti, Chris Drakeley, Inès Vigan-Womas, Makhtar Niang, Henrik Salje, Jessica Vanhomwegen, Michael White

**Affiliations:** 1 Infectious Disease Epidemiology and Analytics Unit, Department of Global Health, Institut Pasteur, Université Paris-Cité, INSERM U1347, Paris, France; 2 Environment and Infectious Risks Unit, Institut Pasteur, Paris, France; 3 Division of Infectious Diseases, Department of Medicine Solna, Karolinska Institutet, Stockholm, Sweden; 4 Department of Infectious Diseases, Södersjukhuset, Stockholm, Sweden; 5 Processus Infectieux en Milieu Insulaire Tropical (PIMIT), Université de La Réunion, INSERM U1187, CNRS 9192, IRD 249, Plateforme Technologique CYROI, Sainte-Clotilde, La Réunion, France; 6 Institut Pasteur, Université Paris Cité, Production and Purification of recombinant Proteins Platform, CNRS UMR3528, Paris, France; 7 International Vaccine Institute, Seoul, Republic of Korea; 8 Department of Virology, Walter Reed Army Institute of Research-Armed Forces Research Institute of Medical Sciences (AFRIMS), Bangkok, Thailand; 9 Philippines-AFRIMS Virology Research Unit, Cebu City, Philippines; 10 Department of Epidemiology, Johns Hopkins Bloomberg School of Public Health, Baltimore, Maryland, United States of America; 11 International Centre for Diarrhoeal Disease Research, Bangladesh (icddr, b), Dhaka, Bangladesh; 12 Department of Pathology and Center for Vector-Borne and Zoonotic Diseases, University of Texas, Medical Branch, Galveston, Texas, United States of America; 13 Department of Microbiology and Immunology, University of Texas Medical Branch, Galveston, Texas, United States of America; 14 MRC Centre for Global Infectious Disease Analysis, School of Public Health, Imperial College London, London, United Kingdom; 15 Department of Infection Biology, Faculty of Infectious and Tropical Diseases, London School of Hygiene and Tropical Medicine, London, United Kingdom; 16 Institut Pasteur de Dakar, Dakar, Senegal; 17 Department of Genetics, University of Cambridge, Cambridge, United Kingdom; Faculty of Science, Ain Shams University (ASU), EGYPT

## Abstract

Arboviruses such as dengue virus (DENV) and chikungunya virus (CHIKV) pose major global health threats. To support serological surveillance and study cross-reactivity, an in-house multiplex bead-based immunoassay was developed to measure IgG responses against a wide panel of orthoflavivirus and alphavirus antigens. Samples from Senegal, France, the Philippines, and Bangladesh enabled analyses to be carried out in endemic and non-endemic regions. IgG kinetics following DENV1 and CHIKV infections revealed virus-specific temporal profiles and identified antigens, such as CHIKV E2 and VLP, as well as DENV1 EDIII and SNAP-tagged EDIII, as promising markers for serosurveillance. A two-step Random Forest model was used to classify PRNT-positive samples and predict PRNT titers. CHIKV showed a strong correlation between IgG and PRNT titers (R² = 0.71), while DENV1 showed weaker performance (R² = 0.42). These results support the use of multiplex serology for arbovirus surveillance and highlight the limitations of using binding antibodies to predict neutralisation, particularly for orthoflaviviruses.

## Author summary

Arboviruses such as dengue and chikungunya are transmitted by mosquitoes and affect millions of people worldwide. In many regions, several viruses circulate at the same time. Detecting past infections using blood tests is essential for understanding how widely these viruses spread, but this is complicated because antibodies against related viruses often cross-react. In this study, we developed a high-throughput blood test that can measure antibodies against many mosquito-borne viruses simultaneously using a very small amount of blood. We applied this approach to samples collected in four countries with different patterns of arbovirus circulation: Senegal, Bangladesh, the Philippines, and France. By analysing antibody responses after confirmed dengue and chikungunya infections, we identified specific viral components that help distinguish true infections from cross-reactive responses. We used machine-learning methods to test whether antibody measurements could predict how antibodies in blood can neutralize viruses. This approach worked well for chikungunya but was less accurate for dengue, reflecting the complex immunity to closely related viruses. Overall, our results show that multiplex antibody testing can improve arbovirus surveillance, especially where several viruses are circulating at the same time.

## Introduction

Arthropod-borne viruses (arboviruses) are transmitted to humans and other vertebrates by blood-feeding arthropods, such as mosquitoes, flies, or ticks. Arboviruses of most human impact are primarily spread by *Aedes spp.* mosquitoes, particularly *Aedes aegypti* and *Aedes albopictus* [1]. Globally, more than 500 arboviruses have been identified, of which 150 are known to cause disease in humans. Arboviruses belong to several families, including *Flaviviridae* (e.g., the orthoflaviviruses dengue (DENV), Zika (ZIKV), yellow fever (YFV), Usutu (USUV), West Nile (WNV), japanese encephalitis viruses (JEV)), and *Togaviridae* (e.g., the *alphaviruses* chikungunya (CHIKV), Mayaro (MAYV), Ross River (RRV), and O’nyong nyong (ONNV) viruses). Dengue virus remains the most prevalent, with 14 million cases and 10,000 deaths reported in 2024 [2]. Chikungunya virus caused 480,000 reported cases and 200 reported deaths in the same year [3].

Although the major Zika virus epidemic occurred in 2016 [4], particularly in Brazil [5] and over 50 other countries worldwide, sporadic and localized cases continue to be reported, indicating a persistent but reduced public health threat [6].

Understanding the circulation of arboviruses within human populations is essential for guiding public health interventions, informing vaccine development and vaccination strategies, and enhancing outbreak preparedness. Seroprevalence studies, which assess the presence of virus-specific antibodies, provide crucial insights into past exposure, immunity levels, and transmission dynamics. These studies play a key role in estimating the true burden of arboviral infections, identifying high-risk populations, and assessing the potential for future outbreaks. However, conducting seroprevalence studies presents several challenges, particularly in regions where multiple arboviruses co-circulate [7].

One of the main challenges in arbovirus serology is the high degree of antibody cross-reactivity among orthoflaviviruses. Due to their structural similarities, antibodies elicited by one virus, such as dengue virus, can cross-react with another, such as Zika or West Nile virus, leading to false-positive results and complicating accurate serodiagnosis. Furthermore, original antigenic sin may further influence immune responses, as prior exposure to one orthoflavivirus affects subsequent responses to related viruses, including memory-driven increases in antibody levels to the first related virus to infect a person [8,9]. Although some degree of cross-reactivity can occur among other arbovirus groups, such as alphaviruses, it is typically more limited [10].

Traditional serological assays, including enzyme-linked immunosorbent assays (ELISA), remain widely used for detecting arboviral antibodies. IgM capture ELISA is typically employed for early infection detection, while indirect IgG assays are used to assess long-term immune responses [11]. However, these assays often lack specificity, particularly in endemic regions with multiple co-circulating arboviruses. Other approaches, such as plaque reduction neutralization tests (PRNTs), often offer greater specificity by measuring virus-neutralizing antibodies. Despite their reliability, PRNTs are labor-intensive, and sometimes require biosafety level 3 (BSL-3) containment, limiting their accessibility, particularly in resource-limited settings [12]. Rapid diagnostic tests (RDTs) have emerged as potential alternatives, but their sensitivity and specificity remain variable, necessitating further validation for widespread implementation [13].

There is a critical need for improved serosurveillance tools that offer greater specificity, sensitivity, and scalability. Multiplex bead-based assays have emerged as a promising option for arbovirus serology, addressing many of the limitations of conventional techniques [14–20]. Unlike conventional ELISA or PRNT, multiplex assays enable the simultaneous detection of antibodies against multiple pathogens in a single reaction, significantly reducing the time, labor, and costs associated with testing as well as the volume of serum or plasma needed. This high-throughput approach enhances the ability to reconstruct exposure history, making it particularly valuable in regions where multiple arboviruses co-circulate. Additionally, by using carefully selected/ engineered recombinant viral antigens and optimized coupling strategies, multiplex assays can improve specificity and minimize cross-reactivity, thereby increasing the reliability of seroprevalence studies. The development and implementation of such advanced serological tools are essential for strengthening arbovirus surveillance and improving outbreak preparedness worldwide. Here, we systematically test a large panel of antigens across several countries and antibody isotypes, compare the results to PRNT values, and explore antibody kinetics following PCR-confirmed infection.

## Materials and methods

### Ethics statement

The Bangladesh study was approved by the ethical review board at icddr,b (protocol #PR-14058) and Institut Pasteur (protocol #2015–074). The Senegalese samples were collected as part of the ongoing Dielmo and Ndiop project, which was approved by the Senegalese National Health Research Ethics Committee (CNERS Sénégal). Approval to measure antibodies in these samples was granted by CNERS Sénégal (N 00000007 MSAS/CNERS/Sec). The Philippines study was approved by the ethical review boards at Vicente Sotto Memorial Medical Center in Cebu City, Philippines, and Walter Reed Army Institute of Research Human Subjects Protection Branch (protocol #1833). Negative samples were obtained from the COVID-Oise study, which was registered with ClinicalTrials.gov (NCT04644159) and received ethical approval from the Comité de Protection des Personnes Nord Ouest IV. Informed consent was obtained from all study participants. For children, informed consent was provided by a parent/guardian.

### Samples

This study utilized panels of samples collected from multiple countries (Table 1).

**Table 1:** Overview of participant and sample data, stratified by country.

country	expected arbovirus exposure	number of participants	number of samples	median age [range]	male (%)	female (%)
Bangladesh	DENV, JEV and CHIKV	78	78	26.5 [6–84]	52.6	47.4
France	non-endemic (control)	84	84	82.5 [8–101]	19	81
Philippines	DENV, ZIKV and CHIKV	36	215	9.5 (1-49)	49	51
Senegal	DENV, ZIKV, YFV, WNV, CHIKV and ONNV	270	270	11.2 [0.3–77]	43.7	51.9

#### Cross-sectional studies.

We analyzed a subset of 74 samples from a cross-sectional seroprevalence study conducted in 2014 in the Chapai Nawabganj district (called zila) of Bangladesh, located in the Rajshahi division. These samples were a subset of 1455 samples collected from 40 communities in the district. In this subset, DENV and CHIKV PRNT testing was conducted in addition to multiplex testing [21].

The Senegalese villages of Dielmo and Ndiop have been monitored longitudinally since the 1990’s with repeat cross-sectional studies to investigate malaria epidemiology [22]. We analysed 270 samples from cross-sectional seroprevalence studies in these villages with samples collected in 2016 and 2018.

#### Longitudinal study.

We analyzed 215 samples from 36 individuals from a longitudinal cohort study conducted in the village of Punta Princesa, located in the Visayas region of the Philippines [14,15]. Briefly, study participants had annual study visits including blood collection at baseline, 12 months, and 24 months. Active fever surveillance was conducted through weekly face-to-face or telephone contacts; any history of fever identified triggered an acute and 3-week convalescent visit with blood collection. Acute serum samples were tested by two-step nested reverse transcriptase PCR (RT-PCR) to detect CHIKV, DENV 1–4, or ZIKV RNA. All samples were obtained from individuals with RT-PCR–confirmed symptomatic infections: ZIKV (N = 1), CHIKV (N = 12), DENV-1 (N = 12; only 10 were included in the analysis because they had complete 3-year follow-up), DENV-2 (N = 6), DENV-3 (N = 2), and DENV-4 (N = 3). These samples were selected for multiplex testing. This allowed us to track longitudinal serological patterns in PCR-confirmed infections.

#### Negative controls.

As negative controls, we also analyzed 84 samples from France, obtained from a longitudinal study on SARS-CoV-2 conducted in Crépy-en-Valois in 2021 [23].

### Antigens

We developed a multiplex bead-based assay comprising 44 antigens (Table 2) from commonly circulating arboviruses, including dengue, Zika, and chikungunya. Recombinant proteins were sourced from Native Antigen Company (Oxford, UK), Cusabio (Houston, USA), Alpha Diagnostics Intl. Inc. (San Antonio, USA) or Clinisciences (Nanterre, France).

**Table 2:** List of antigen names and suppliers used for multiplex bead-based assay development.

Antigen name	Short name	Strain/ Isolate Information	Supplier	Family	Catalogue number
Chikungunya soluble ectodomain E2	CHIKV E2	Senegal 37997	Native antigen	*Togaviridae*	REC31617–100
Chikungunya virus-like particles	CHIKV VLP	Senegal 37997	Native antigen	*Togaviridae*	CHIKV-VLP-100
Chikungunya non structural protein	CHIKV NSP	strain S27-African prototype	Cusabio	*Togaviridae*	CSB-EP810351CJAT
SNAP-chikungunya soluble ectodomain E2	CHIKV E2 + SNAP	06-049	In-house production – EVAg	*Togaviridae*	014P-03540
Dengue 1 domain III envelope protein	DENV1 EDIII	Dengue virus 1 Nauru/West Pac/1974	Native antigen	*Flaviviridae*	REC32042–100
Dengue 1 non structural protein 1	DENV1 NS1	Nauru/Western Pacific/1974	Native antigen	*Flaviviridae*	DENV1-NS1–100
Dengue 1 virus-like particles	DENV1 VLP	Puerto Rico/US/BID-V853/1998	Native antigen	*Flaviviridae*	DENVX4-VLP-100
Dengue 2 domain III	DENV2 EDIII	Dengue virus 2 Jamaica/1409/1983	Native antigen	*Flaviviridae*	REC32043–100
envelope protein					
Dengue 2 non structural protein 1	DENV2 NS1	Thailand/16681/84	Native antigen	*Flaviviridae*	DENV2-NS1–100
Dengue 2 virus-like particles	DENV2 VLP	Thailand/16681/84.	Native antigen	*Flaviviridae*	DENVX4-VLP-100
Dengue 3 domain III envelope protein	DENV3 EDIII	Dengue virus 3 isolate 15/1329	Native antigen	*Flaviviridae*	REC32044–100
Dengue 3 non structural protein 1	DENV3 NS1	Sri Lanka D3/H/IMTSSA-SRI/2000/1266	Native antigen	*Flaviviridae*	DENV3-NS1–100
Dengue 3 virus-like particles	DENV3 VLP	Sri Lanka D3/H/IMTSSA-SRI/2000/1266	Native antigen	*Flaviviridae*	DENVX4-VLP-100
Dengue 4 domain III envelope protein	DENV4 EDIII	DENV-4/SG/06K2270DK1/2005	Native antigen	*Flaviviridae*	REC32045–100
Dengue 4 non structural protein 1	DENV4 NS1	Dominica/814669/1981	Native antigen	*Flaviviridae*	DENV4-NS1–100
Dengue 4 virus-like particles	DENV4 VLP	Dominica/814669/1981	Native antigen	*Flaviviridae*	DENVX4-VLP-100
SNAP-Dengue 1 domain III envelope protein	DENV1 EDIII + SNAP	FGA/NA d1d	In-house production – EVAg	*Flaviviridae*	014P-03514
SNAP-Dengue 2 domain III envelope protein	DENV2 EDIII + SNAP	CS85–3	In-house production – EVAg	*Flaviviridae*	014P-03515
SNAP-Dengue 3 domain III envelope protein	DENV3 EDIII + SNAP	DENV-3/TH/BID-V2329/2001	In-house production – EVAg	*Flaviviridae*	014P-03516
SNAP-Dengue 4 domain III envelope protein	DENV4 EDIII + SNAP	CS101-1	In-house production – EVAg	*Flaviviridae*	014P-03517
Japanese encephalitis envelope protein	JEV E	SA-14	Native antigen	*Flaviviridae*	REC31688–100
Japanese encephalitis non structural protein 1	JEV NS1	SA-14	Native antigen	*Flaviviridae*	JEV-NS1–100
SNAP Japanese encephalitis domain III envelope protein	JEV EDIII + SNAP	GP05	In-house production – EVAg	*Flaviviridae*	014P-03521
Mayaro non structural protein 1	MAYV NSP	Strain Brazil	Alpha Diagnostics Intl. Inc.	*Togaviridae*	AD-MAYV41-R-25
Mayaro soluble ectodomain E2	MAYV E2	Acre27	Native antigen	*Togaviridae*	REC31644–100
SNAP-Mayaro soluble ectodomain E2	MAYV E2 + SNAP	Brazil BeH256	In-house production – EVAg	*Togaviridae*	014P-03542
O’nyong nyong soluble ectodomain E2	ONNV E2	SG650	Clinisciences	*Togaviridae*	IT-022–006Ep
O’nyong nyong virus-like particles	ONNV VLP	SG650	Native antigen	*Togaviridae*	REC31626–100
Ross River structural polyprotein	RRV SP	T48	Native antigen	*Togaviridae*	REC31969–100
SNAP Ross River soluble ectodomain E2	RRV E2 + SNAP	QML1	In-house production – EVAg	*Togaviridae*	014P-03541
Rift Valley fever nucleoprotein	RVFV NP	ZH-548	Native antigen	*Phenuiviridae*	REC31640–100
Usutu non structural protein 1	USUV NS1	Vienna 2001	Native antigen	*Flaviviridae*	USUV-NS1–100
SNAP Usutu domain III envelope protein	USUV EDIII + SNAP	Usu973	In-house production – EVAg	*Flaviviridae*	017P-06227
West Nile domain III envelope protein	WNV EDIII	lineage 1, strain NY99	Sino Biological	*Flaviviridae*	40345-V08Y
West Nile non structural protein 1	WNV NS1	NY99	Native antigen	*Flaviviridae*	WNV-NS1–100
SNAP West Nile domain III envelope protein	WNV EDIII + SNAP	DES 1476-01	In-house production – EVAg	*Flaviviridae*	014P-03519
Yellow fever envelope protein	YFV E	17D-204-USA vaccine, isolate HONG3	Native antigen	*Flaviviridae*	REC32081–100
Yellow fever non structural protein 1	YFV NS1	17D	Native antigen	*Flaviviridae*	YFV-NS1–100
SNAP Yellow fever domain III envelope protein	YFV EDIII + SNAP	Asibi	In-house production – EVAg	*Flaviviridae*	014P-03520
Zika Uganda strain non structural protein 1	ZIKV U.NS1	Uganda MR 769	Native antigen	*Flaviviridae*	ZIKV-NS1–100
Zika Asian strain domain III envelope protein	ZIKVAS EDIII	Zika virus isolate Z582-16 (Brazil, March 2018)	Native antigen	*Flaviviridae*	REC32046–100
Zika Suriname strain non structural protein 1	ZIKV Su. NS1	Suriname Z1106033	Native antigen	*Flaviviridae*	ZIKVSU-NS1–100
SNAP-Zika domain III envelope protein	ZIKV EDIII + SNAP	EC YAP	In-house production – EVAg	*Flaviviridae*	014P-03522
SNAP control	SNAP	–	In-house production – EVAg	*Control*	–

In parallel, we utilized recombinant orthoflaviviruses envelope domain III (EDIII) and soluble ectodomains of alphaviruses envelope (E2) protein prepared at Institut Pasteur [24] (Table 2). These recombinant viral antigens were expressed as chimeric proteins fused to a modified O6-alkylguanine-DNA alkyltransferase enzyme (AGT), referred to as the SNAP domain. The SNAP-antigen fusion proteins were coupled to the microspheres using an oriented, two-step coupling procedure to ensure optimal antigen presentation.

### Manual-oriented coupling of in-house recombinant proteins

Manual coupling of recombinant proteins containing the SNAP-tag was performed using the Bio-Plex Amine Coupling Kit (Bio-Rad). The first step consists of coupling the AGT substrate BG-PEG-NH_2_ to the activated microspheres by amine coupling, and the second step consists of binding the SNAP-tagged proteins to the substrate-coated microspheres.

The stock of uncoupled microspheres was sonicated and vortexed for 30 seconds. Subsequently, 2.5 × 10⁶ microspheres (200 µL) were transferred to an Eppendorf tube and washed once with bead wash buffer using a magnetic separator. The beads were then activated for 20 minutes at room temperature (RT) with 80 µL of bead activation buffer, 10 µL of hydroxysulfosuccinimide sodium salt (S-NHS; 50 mg/mL, Sigma-Aldrich), and 10 µL of 1-ethyl-3-(3-dimethylaminopropyl)carbodiimide hydrochloride (EDAC; 50 mg/mL, Sigma-Aldrich). Both S-NHS and EDAC were prepared in bead activation buffer immediately prior to use.

Following activation, the beads were washed twice with Phosphate Buffer Saline buffer (PBS), and 5 mg/mL of BG-PEG-NH_2_ was added to the coupling reaction. The beads were incubated overnight at RT on a shaker. After incubation, the beads were washed twice with PBS buffer and resuspended in 250 µL of blocking buffer. This suspension was incubated for 30 minutes in the dark on a shaker.

After blocking, the beads were washed twice with PBS, and 50–100 µg of recombinant proteins was added in 1 mL of PBS. The optimal amount of recombinant proteins (µg) per 1.25x10^6^ of beads was optimised in a previous study, using an anti-SNAP polyclonal antibody (New England Biolabs) to obtain a saturation of the substrate BG-PEG-NH_2_ immobilised on the surface of the microspheres. The suspension was incubated in the dark on a roller for 2 hours. The coupled beads were then washed twice with PBS and resuspended in 1 mL of storage buffer. The beads were stored at 4°C.

One day after the coupling process, the antigen-coupled beads were verified by using an anti-SNAP polyclonal antibody, and the results were compared with those from the optimisation process to confirm coupling efficiency. After verification, all coupled beads were counted using the TC20 Automated Cell Counter (Bio-Rad).

### Automated coupling procedure

For recombinant proteins that do not contain the SNAP-tag, the coupling procedure was automated using the KingFisher Duo Prime magnetic particle processor (Thermo Fisher Scientific, Waltham, MA, USA). This instrument is equipped with a 12-well magnet head and 96-deep well plates. The protocol implemented in the processor was adapted from the manual coupling procedure [25]. A detailed description of the BindIt software protocol (version 4.0.0.45) is provided in the supplementary data (Methods in S2 Appendix).

This automated method allowed for the coupling of up to 12 recombinant proteins in a single run. Buffers, volumes, and incubation times were adjusted to fit the automated workflow. For each run, the instrument was set up with a bead comb and two deep-well plates (Thermo Fisher Scientific) containing the necessary buffers and microspheres, as specified by the program. The composition and layout of the two plates are detailed in the supplementary methods (Table A in S1 Appendix).

Once the automated run was completed, the coupled beads were transferred from the 96-well plate to Eppendorf tubes. The beads were stored at 4°C, and one day after the coupling process, the antigen-coupled beads were verified using a pool of serum from arbovirus-exposed individuals to assess whether they generated a log-linear standard curve. Then, the coupled beads were counted using the TC20 Automated Cell Counter (Bio-Rad).

### Multiplex immunoassay (MIA)

The bead-based multiplex immunoassay (MIA) procedure was performed as previously described [25] with some adjustments. Briefly, a mixture of 44 antigen-coupled bead regions (1,000 beads/region/well) was sequentially incubated in the dark under constant shaking with diluted serum samples: 1:400 for IgG and 1:200 for IgA and IgM. The incubation was carried out for 30 minutes on a shaker. All serum dilutions and bead premixes were prepared in PBT buffer (phosphate-buffered saline containing 1% bovine serum albumin and 0.05% (v/v) Tween-20).

Following incubation, the plates were washed three times with PBS 1X containing 0.05% Tween-20 using a HydroSpeed plate washer (Tecan). After washing, the plates were incubated for 15 minutes with a secondary antibody conjugated to R-phycoerythrin (2 µg/mL) specific for IgG, IgA, or IgM (Jackson Immunoresearch, UK). The plates were then washed three more times using the HydroSpeed plate washer and resuspended in 150 µL of PBS 1X with 0.05% Tween-20. Plates were read using the Intelliflex system at a low Photomultiplier Tube (PMT) setting, and the median fluorescence intensity (MFI) was measured.

Samples were run on 96-well plates, containing a blank (only beads, without serum) and a standard curve prepared from two-fold serial dilutions (1:50–1:102 400) of a pool of serum from arbovirus-exposed individuals. A normalised MFI (nMFI) for the antibody response to an antigen in a sample was calculated by dividing the measured MFI by the MFI of the 1:400 dilution of the pool of serum from arbovirus-exposed individuals. For antigens containing a SNAP tag, the nMFI was calculated by dividing the measured MFI in a sample by the MFI to the SNAP tag only in the same sample.

The protocol for measuring IgG avidity is detailed in the S1 Appendix, and results are shown in Figs E,H,L, Q, U and Z in S1 Appendix.

### Plaque reduction neutralisation test (PRNT)

Plaque reduction neutralisation tests (PRNT) were performed using the procedure of Russell et *al.*[26] and others [27]. In brief, four-fold serial dilutions of sera from 1:10 were incubated with attenuated CHIKV strain 181/clone25 derived from a CHIKV Asian strain isolated from Thailand in 1962 [28]. Mixtures of virus-serum were incubated on a monolayer of Macaca mulatta kidney (LLC-MK2) cells in a 12-well plate with 30–50 plaques in control wells. PRNT titers were expressed as the extrapolated serum dilution causing 80% plaque reduction. DENV PRNTs in Bangladesh were conducted by the Vasilakis laboratory at UTMB as described previously [29].

### Comparison with a commercially available serological assay

To further characterize assay performance, a direct comparison was performed between the multiplex bead-based immunoassay and a commercially available serological kit from Tetracore (TC-Custom-096). This assay provides the qualitative detection of IgG antibodies to nine different arboviruses in human serum (ZIKV, DENV 1–4, JEV, WNV, YFV, and CHIKV). The two assays were applied to an identical subset of serum samples (n = 42). Serum samples were diluted for the Tetracore assay at 1/500 and for our in-house assay at 1/400, and results were analyzed to assess concordance (Fig A in S1 Appendix).

### Random forest models for predicting arboviral PRNTs using multiplex serological data

We used a two-step random forest modelling approach to assess whether the multiplex serological data could be used to predict PRNT titers for DENV (Serotype 1–3, Figs M and R in S1 Appendix) and CHIKV in Bangladesh. We did not fit models to DENV serotype 4 because of low rates of positivity in the tested samples. The model was fitted separately to PRNT data for each DENV serotype and CHIKV.

In step 1 (Classification), a Boruta feature selection algorithm [30] was used to identify all serological responses that contributed significant information for classification of PRNT status (i.e., positive or negative). A random forest classifier was then fitted to the reduced set of serological predictors to estimate the probability of PRNT positivity for each sample, and a fixed probability threshold of 0.5 was used to define predicted PRNT status.

Step 2 (Regression) was applied only to samples that were predicted to be PRNT positive in step 1. In this step, a second Boruta feature selection was performed to identify all serological responses informative for predicting PRNT titer, and a random forest regression model was then fitted to the reduced set of predictors to estimate the PRNT titer in PRNT positive samples.

Hyperparameters were tuned separately for each step by cross-validation. Model performance was assessed using out-of-bag (OOB) predictions, defined as predictions for each sample based only on trees for which the sample was not included in the bootstrap resample. For step 1 we used OOB class probabilities, and for step 2 we used OOB predicted titers. The distribution of tree-level OOB predictions was used to derive prediction intervals. Because feature selection and tuning were performed on the available dataset for each outcome, OOB performance should be interpreted as internal validation.

Predictions from both steps were aggregated to obtain a final PRNT titer estimate for each sample, which was compared with the actual observed PRNT titer to assess model performance.

All analyses were performed using R (version 4.4.3).

## Results

### Distribution of IgG antibody for alphaviruses across four countries

Fig 1 illustrates the distribution of IgG nMFI against alphaviruses antigens from our 44-plex assay in a total of 647 samples from four countries (France, Bangladesh, Senegal and the Philippines). Overall, serological responses varied across countries, suggesting differences in exposure to alphaviruses. The correlation between measured antibody responses is presented in Fig B in S1 Appendix.

**Fig 1:**
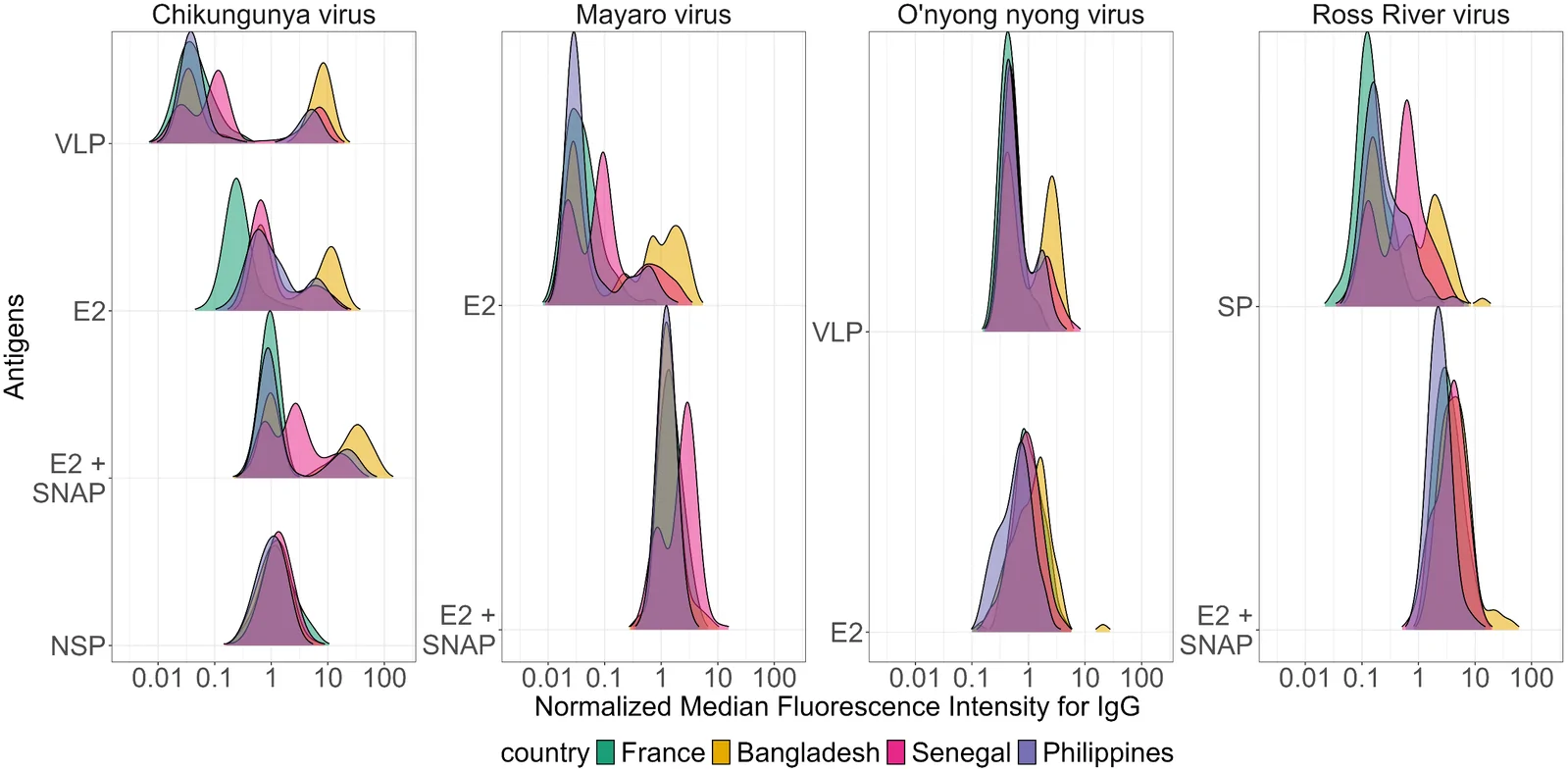
Distribution of IgG antibody responses for alphaviruses. Normalized Median Fluorescence Intensity (nMFI) for IgG responses against antigens from chikungunya, Mayaro, O'nyong-nyong, and Ross River viruses in samples from France (green), Bangladesh (yellow), Senegal (pink), and the Philippines (purple). nMFI values are derived from median fluorescence intensity (MFI) using a Luminex INTELLIFLEX assay. The antigens assessed include virus-like particles (VLP), envelope glycoprotein E2 (E2), SNAP-tagged E2 (E2 + SNAP), non-structural protein (NSP) for chikungunya virus, and structural polyprotein (SP) for Ross River virus.

A strong chikungunya IgG response was observed in samples from Bangladesh, Senegal and the Philippines, particularly against VLP, E2 and E2 + SNAP antigens. The presence of a clear bimodal distribution suggests widespread prior exposure in these regions. In contrast, samples from France displayed low IgG responses across all CHIKV antigens, consistent with limited and localized CHIKV transmission reported in southern France [31,32].

There is no clear signal for CHIKV NSP suggesting limited utility for serodiagnosis in this context. Concerning MAYV, ONNV and RRV viruses there was evidence of bi-modal distributions for MAYV E2, ONNV VLP and RR SP, but there is currently no publish evidence of these viruses circulating in Bangladesh, the Philippines or Senegal. This could be explained by cross-reactivity with other circulating alphaviruses such as CHIKV.

### Distribution of IgG antibody for orthoflaviviruses across four countries

To assess serological responses to orthoflaviviruses we analyzed IgG antibody levels against nine orthoflaviviruses across four countries (Fig 2). We observed a strong dengue IgG responses in samples from Bangladesh, Senegal and the Philippines, particularly against the non structural protein 1 (NS1) and virus-like particles (VLP) with evidence of bimodal distributions. In contrast, samples from France showed lower IgG levels, consistent with the near absence of endemic DENV tranmission. EDIII and EDIII + SNAP signals showed a smaller dynamic range compared to VLP and NS1.

**Fig 2:**
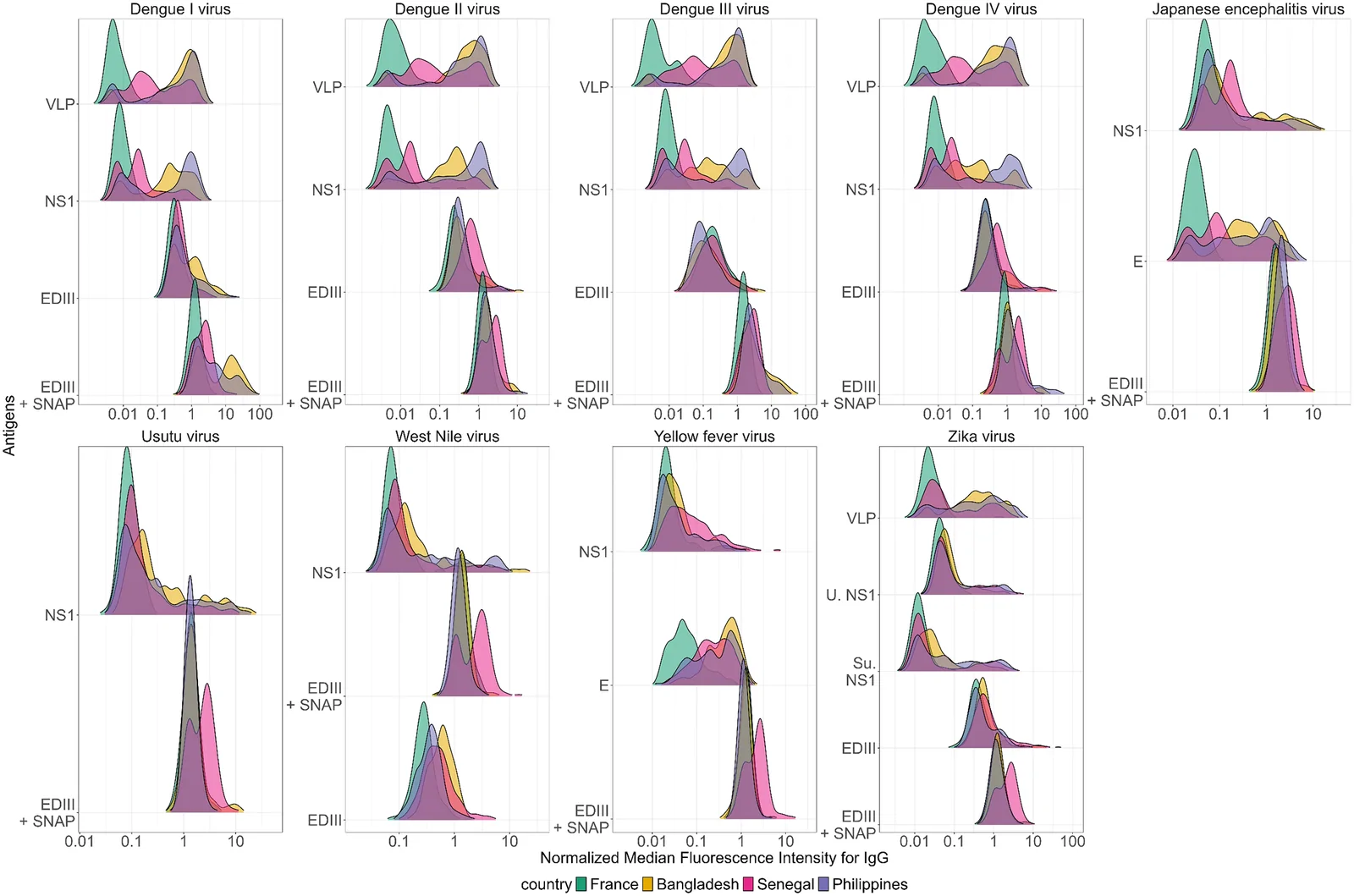
Distribution of IgG antibody responses for orthoflaviviruses. Normalized Median Fluorescence Intensity (nMFI) for IgG responses against antigens from dengue (serotypes 1-4), Japanese encephalitis, usutu, West-Nile, yellow fever and Zika viruses in samples from France (green), Bangladesh (yellow), Senegal (pink) and the Philippines (purple). nMFI values are derived from median fluorescence intensity (MFI) using a Luminex INTELLIFLEX assay. The antigens assessed include virus-like particles (VLP), non-structral protein (NS1), envelope (E), envelope domain III (EDIII), SNAP-tagged envelope domain III (EDIII + SNAP), and NS1 proteins from Uganda strains (U. NS1) and Suriname strain (Su. NS1) for Zika virus.

### Antibody kinetics following chikungunya infection

We analyzed samples from a study conducted in the Philippines where individuals were followed longitudinally for up to 3 years after RT-PCR confirmation of infection with arboviruses including CHIKV, ZIKV, and DENV1–4 (Figs I, N, S and W in S1 Appendix). Firstly, a notable increase in antibody levels is observed post-infection. CHIKV-specific antigens are highly immunogenic, leading to strong IgG responses with large boosts in antibody levels (Fig 3). Secondly, antibody responses after CHIKV infection are long-lived responses especially for VLP, E2 and E2 + SNAP. CHIKV infection caused substantial boosting of IgG responses against other alphaviruses, providing strong evidence of cross reactivity.

**Fig 3:**
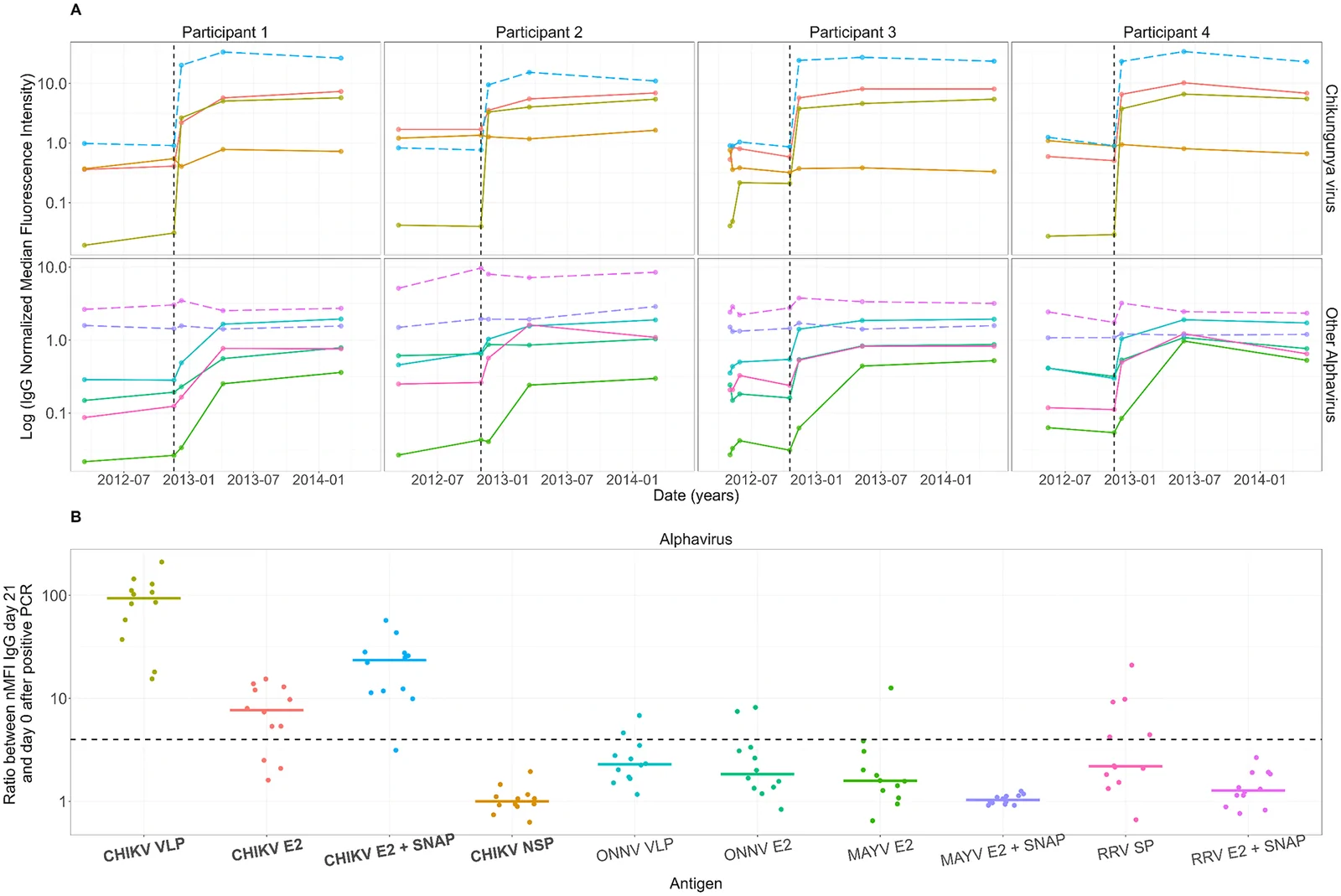
Alphavirus antibody kinetics in individuals with RT-PCR-confirmed CHIKV infection. (A) Longitudinal IgG antibody responses to CHIKV and other alphaviruses in 4/12 representative infected individuals. Each column represents an infected individual (participants 1 to 4), with the upper row showing responses to CHIKV-specific antigens (NSP, E2, VLP, and SNAP + E2) and the lower row displaying responses to other alphavirus antigens. The dashed vertical line indicates the date of RT-PCR-confirmed CHIKV infection. Antigens represented with dashed lines correspond to SNAP antigens. (B) Alphavirus antibody boost in RT-PCR-confirmed CHIKV infections. The nMFI IgG ratio between day 21 and day 0 after RT-PCR-confirmed CHIKV infection is presented. The dashed dotted line represents a 4-fold increase in nMFI. Each antigen is represented by a unique colour, defined in panel B.

### Antibody kinetics following DENV1 virus infection

Fig 4 presents the longitudinal IgG antibody responses to DENV and other orthoflaviviruses antigens in four DENV1 RT-PCR-positive participants. In all individuals, a marked and rapid increase in IgG levels was observed against DENV1-specific antigens following infection, indicating a robust humoral immune response. IgG responses increased after infection and were followed by either sustained high levels or a gradual decline over time.

**Fig 4:**
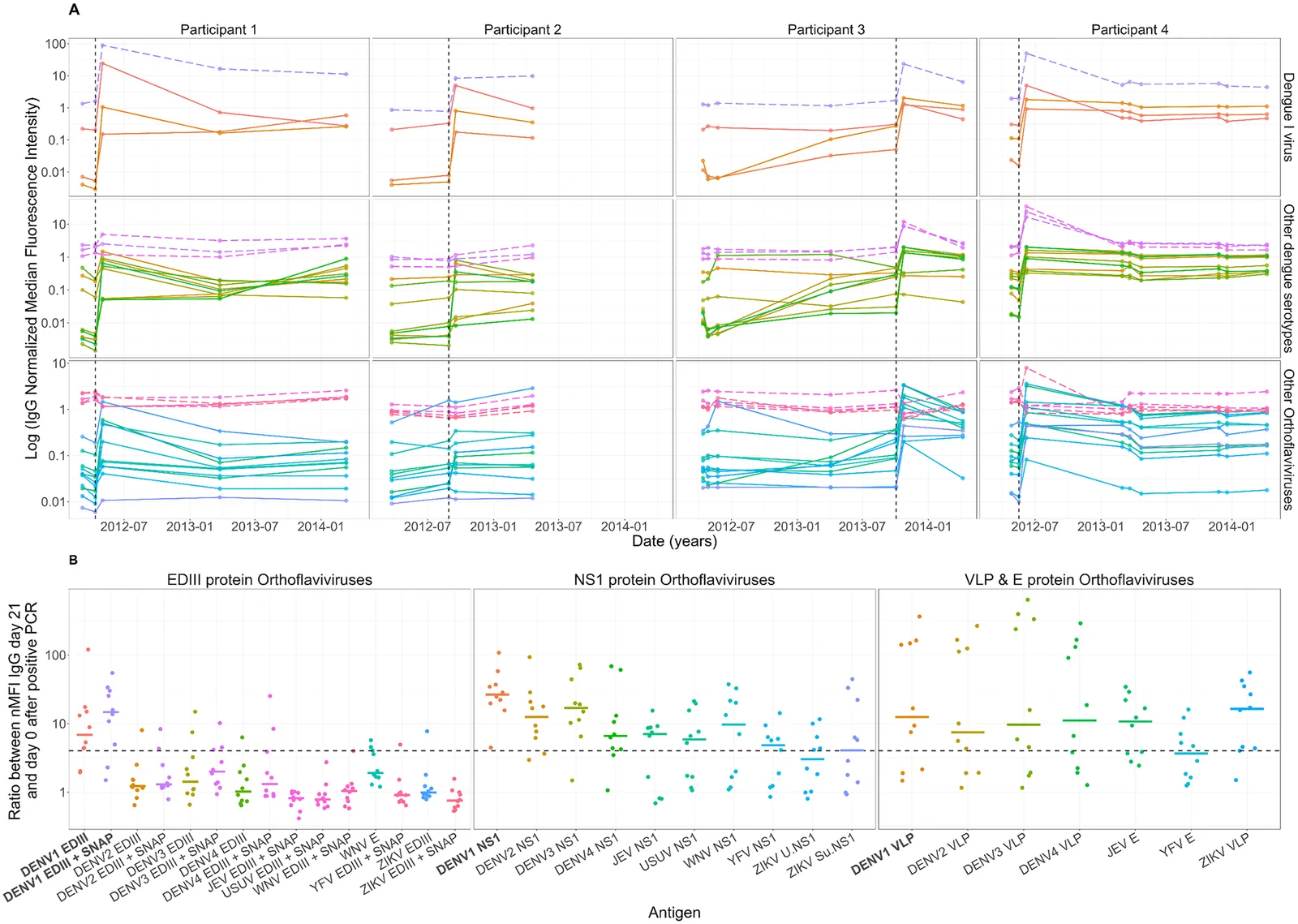
Orthoflaviviruses antibody kinetics in individuals with RT-PCR-confirmed DENV1 infection. (A) Longitudinal IgG antibody responses to DENV1 and other orthoflaviviruses in 4/10 representative infected individuals. Each column represents an infected individual (participants 1 to 4), with the top row showing responses to DENV1-specific antigens (VLP, NS1, EDIII and EDIII + SNAP), the second row showing responses to DENV2,3,4 specific antigens, and the third row displaying responses to other orthoflaviviruses antigens. The dashed vertical line indicates the date of RT-PCR-confirmed DENV1 infection. Antigens represented with dashed lines correspond to SNAP antigens. (B) Orthoflaviviruses antibody boost following RT-PCR-confirmed DENV1 infection. The nMFI IgG ratio between day 21 and day 0 after RT-PCR-confirmed DENV1 infection is presented. The dashed dotted line represents a 4-fold increase in nMFI. Each antigen is represented by a unique colour, consistent across all panels.

DENV1-infection also elicited IgG responses against other orthoflaviviruses, providing evidence of cross reactivity. This was most notable among heterologous DENV serotypes, but evident also with other orthoflaviviruses such as ZIKV. These findings underscore the complexity of orthoflavivirus serological responses. Due to this cross-reactive boosting, accurately identifying the infecting serotype based solely on IgG kinetics remains a notable challenge in endemic areas.

### Comparison of the multiplex serological assay with the Plaque Reduction Neutralisation Test (PRNT) for CHIKV

To compare the serological multiplex assay with the gold standard PRNT for CHIKV, the the association between IgG levels and PRNT titers in samples from Bangladesh was performed. First, we assessed the correlation between PRNT titers and IgG responses to individual antigens (Fig 5).

**Fig 5:**
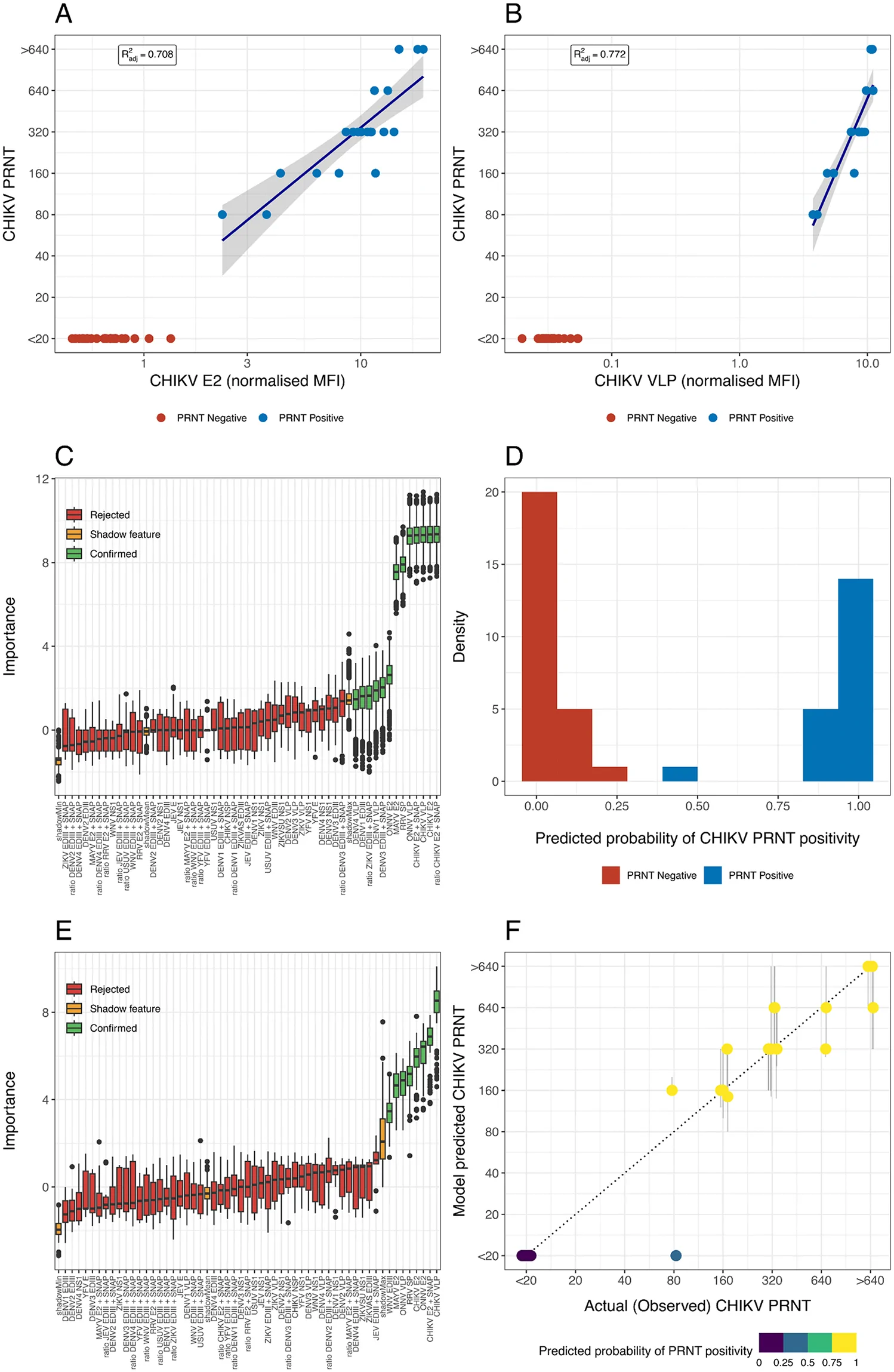
Two-step Random Forest model to predict CHIKV PRNT titer in Bangladesh samples. Comparison of CHIKV PRNT and (A) CHIKV E2 (nMFI) and (B) CHIKV VLP (nMFI). (C) Boruta variable importance for classification of CHIKV PRNT positivity (Step 1). The boxplots represent the distribution of importance scores for the tested antigens. Green boxes represent the features/antibody responses that contributed significantly to classification and were retained for the final classifier. Red boxes represent rejected features that did not provide meaningful classification power. Yellow boxes represent shadow features that serve as controls to determine the threshold for importance. (D) Distribution of the OOB predicted probability of CHIKV PRNT positivity from the random forest classification step (Step 1). PRNT positive samples are shown in blue and PRNT negative samples are shown in red. (E) Boruta variable importance for regression of CHIKV PRNT titers (Step 2). Green boxes represent antibody responses that were retained for the final regression model. (F) Aggregated OOB predictions of CHIKV PRNT titers from the full two-step random forest model (Step 1: classification and Step 2: regression). This figure compares the actual (observed) PRNT titers with model-predicted PRNT titers. Each point represents a sample. Colors indicate the predicted probability of PRNT positivity (ranging from purple [low probability] to yellow [high probability]). The dashed diagonal line represents perfect agreement between observed and predicted PRNT titer. Points clustering along this line indicate accurate predictions, while deviations suggest potential model limitations or outliers.

Fig 5A-5B illustrates the strong linear relationship between IgG responses to CHIKV antigens (E2 and VLP) and PRNT titers in PRNT-positive samples (adjusted R² > 0.7). However, this also illustrates that a simple linear model based on the antibody response to an individual CHIKV antigen would be unable to simultaneously predict PRNT titers in positive individuals and identify PRNT-negative cases.

We therefore applied a two-step random forest model that combines classification and regression to evaluate whether a more flexible model fitted to the multiplex serological data can improve the ability to identify PRNT-negative individuals and accurately predict the PRNT titers across the full range of values (Fig 5C–5F). The model showed strong performance, with most predicted titers closely matching observed titers (points clustering along the diagonal) (Fig 5F). Importantly, samples with low PRNT titers (<20) were reliably identified as negative, while higher titers were accurately estimated.

### Comparison of multiplex serological assay with PRNT assay for DENV1

For DENV1, there was a modest correlation between the PRNT titers and IgG levels to DENV NS1 and VLP antigens in PRNT positive individuals (Fig 6A and 6B). This highlights the challenge of predicting orthoflaviviruses neutralization from antibody binding data alone. To explore whether multiplex IgG profiles could be used to predict PRNT outcomes, we applied the two-step Random Forest algorithm (Fig 6C–6F). The model showed limited predictive performance both with regards to identifying PRNT positive individuals (Fig 6C–6D) and for predicting PRNT titers in samples classified as PRNT positive (Fig 6E and 6F). These results indicate that predicting PRNT titers for orthoflaviviruses like DENV1 is more challenging, likely due to cross-reactivity and complex immune profiles of the Bangladeshi samples. However, the analysis did identify the most informative antigens at each model step, which included the ratio of DENV1 EDIII + SNAP, DENV1 VLP, DENV2 VLP, DENV4 VLP, and the ratio of DENV3 EDIII + SNAP highlighting the potential diagnostic relevance for these antigens (Fig 6C and 6E).

**Fig 6:**
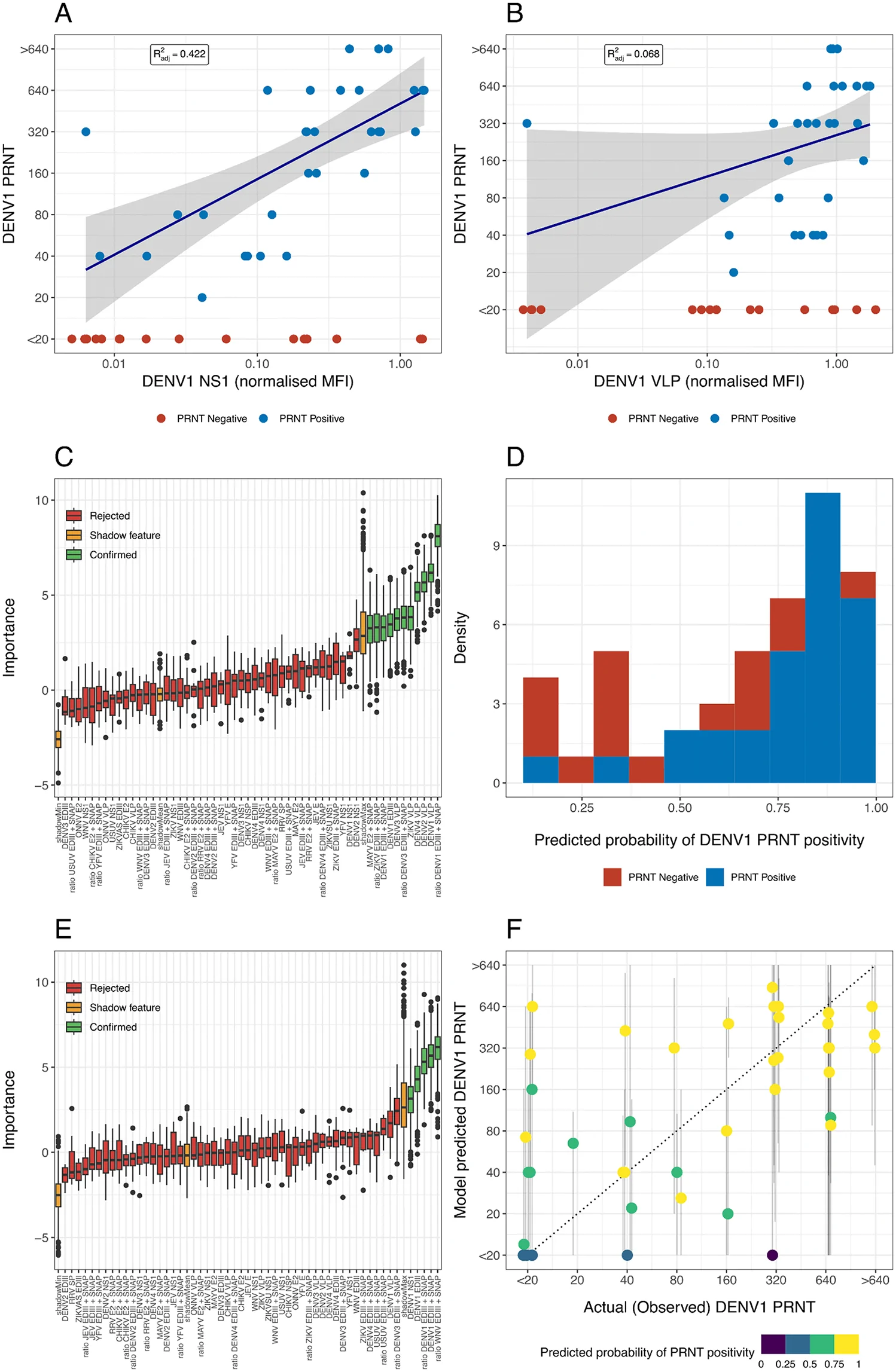
Two-step Random Forest model to predict DENV1 PRNT titer in Bangladesh samples. Comparison of DENV1 PRNT and (A) DENV1 NS1 (nMFI) and (B) DENV1 VLP (nMFI). (C) Boruta variable importance for classification of DENV1 PRNT positivity (Step 1). The boxplots represent the distribution of importance scores for the tested antigens. Green boxes represent the features/antibody responses that contributed significantly to classification and were retained for the final classifier. Red boxes represent rejected features that did not provide meaningful classification power. Yellow boxes represent shadow features that serve as controls to determine the threshold for importance. (D) Distribution of the OOB predicted probability of DENV1 PRNT positivity from the random forest classification step (Step 1). PRNT positive samples are shown in blue and PRNT negative samples are shown in red. (E) Boruta variable importance for regression of DENV1 PRNT titers (Step 2). Green boxes represent antibody responses that were retained for the final regression model. (F) Aggregated OOB predictions of DENV1 PRNT titers from the full two-step random forest model (Step 1: classification and Step 2: regression). This figure compares the actual (observed) PRNT titers with model-predicted PRNT titers. Each point represents a sample. Colors indicate the predicted probability of PRNT positivity (ranging from purple [low probability] to yellow [high probability]). The dashed diagonal line represents perfect agreement between observed and predicted PRNT titer. Points clustering along this line indicate accurate predictions, while deviations suggest potential model limitations or outliers.

## Discussion

Serological surveillance is a key tool for the major challenge of understanding the spread and immune landscape of arboviruses, particularly in regions where multiple viruses co-circulate. In this study, we applied a high-throughput Luminex-based multiplex immunoassay to assess IgG responses to alphaviruses and orthoflaviviruses across four epidemiologically distinct countries: Bangladesh, the Philippines, Senegal, and France.

Our findings highlight both the potential and limitations of IgG-based serology assays in distinguishing infections with closely related arboviruses, especially given the known cross-reactivity within viral families. IgG is a valuable marker for assessing past exposure, as it typically becomes detectable within a few weeks after infection [33].Comparison of traditional methods with our multiplex bead-based immunoassay revealed substantial advantages such as simultaneous detection of IgG antibodies against 44 different antigens using a single, small-volume serum sample at low cost conserving high specificity and efficiency. Comparison with a commercially available serological kit further supports the performance of the MIA. Importantly, correlations observed between pairwise antigen responses were consistent across both methods, indicating that the multiplex assay reliably captures biologically meaningful antibody patterns (Fig A in S1 Appendix). A key advantage of the in-house MIA, however, is its ability to simultaneously measure responses to multiple antigens for each virus, whereas commercial assays typically target a single representative antigen. This multi-antigen resolution allows for more nuanced interpretation of serological profiles, particularly in settings with high cross-reactivity, by enabling concordant or discordant antigen responses to be assessed within the same individual. By measuring responses to a panel of arboviruses antigens across multiple geographic settings, and testing both longitudinal samples from RT-PCR–confirmed infections and cross-sectional samples (including those with paired neutralization data), we identified markers with good discriminatory power between seropositive and seronegative individuals.

A strong chikungunya IgG response was observed in samples from Bangladesh and Senegal, particularly against VLP, E2, and E2 + SNAP antigens. The presence of a clear bimodal distribution suggests widespread prior exposure in these regions. In contrast, samples from France displayed low IgG responses across all CHIKV antigens, consistent with limited and localized CHIKV transmission reported in southern France [31,32]. The distribution of the IgG response to CHIKV antigens exhibited a clear peak corresponding to infected individuals. For the uninfected individuals, there was substantial variation between populations which we attribute to differences in total IgG levels between populations [34,35]. Similar patterns were observed in the IgG kinetics of RT-PCR–confirmed CHIKV infections, with VLP, E2, and E2 + SNAP responses showing a marked post-infection increase compared with other alphavirus antigens (Fig 3). These antigens show strong potential as markers for distinguishing alphavirus infections. In contrast, responses to the CHIKV NSP antigen were less discriminative, indicating limited diagnostic utility (Figs 1 and 3).

Low-level of ONNV transmission is restricted to Africa [36]. However, the distribution of IgG responses to ONNV was similar between Senegal, where transmission has been documented [32], and Bangladesh and the Philippines, where circulation is not documented. The ONNV VLP antigen showed a response profile similar to CHIKV VLP, suggesting cross-reactivity and limited specificity. In contrast, the ONNV E2 antigen elicited low and consistent IgG levels across all four countries, indicating minimal exposure, and supporting its potential as a specific marker for ONNV seroprevalence. Despite the close phylogenetic relationship between ONNV and CHIKV, cross-reactivity after CHIKV infection was modest and comparable to that observed with MAYV, a more distantly related alphavirus [37] (Fig 3). This was unexpected, and although a slight boost in IgG was observed for ONNV VLP, ONNV E2, MAYV E2 and MAYV E2 + SNAP following CHIKV infection, these antigens still performed well to distinguishing CHIKV from other alphaviruses infections. The inclusion of longitudinal samples from RT-PCR–confirmed cases was critical for identifying the most informative markers, as it enabled evaluation of antigen responses over time, something not possible in cross-sectional studies where past exposure is often uncertain.

Although RRV has not been officially reported in Senegal, France, or Bangladesh, the RRV SP antigen elicited strong IgG responses in participants from Bangladesh. This may reflect cross-reactivity with other circulating alphaviruses, particularly CHIKV, as evidenced by the boost in anti-RRV SP antibodies following RT-PCR-confirmed CHIKV infection (Fig 3). Notably, recent studies suggest possible RRV circulation in parts of the Pacific, highlighting the need for further investigation into its geographic distribution [38,39]. The SNAP-tagged E2 antigen for RRV produced uniformly low IgG signals, highlighting its potential for improved specificity in serodiagnosis. In contrast, for MAYV which is endemic primarily to South and Central America [40], IgG responses to the E2 protein in non-endemic regions likely reflect cross-reactivity rather than true exposure (Fig 1). Again, the SNAP-tagged E2 antigen showed lower IgG responses. This pattern was also evident in Fig 3, where CHIKV infection elicited a boost in IgG against MAYV E2 and MAYV E2 + SNAP, but these increases were less pronounced than those observed for CHIKV antigens, reinforcing its value for reducing cross-reactivity in serological assays [41].

Orthoflaviviruses IgG responses are known to exhibit substantial cross-reactivity, complicating serological differentiation. Among DENV antigens, NS1 and VLP consistently generated the highest IgG levels, indicating their sensitivity for identifying past exposure. However, due to the high degree of correlation of these antibody responses between different orthoflaviviruses, these antigens have limited specificty. Following RT-PCR-confirmed DENV1 infection, the boost to anti-EDIII antibodies was notably smaller than for the VLP and NS1 antigens, indicating that EDIII is a less immunogenic biomarker of infection. However, in contrast to the VLP and NS1 antigens, the boost in anti-EDIII antibodies following infection was much more pronouced for the infecting DENV serotype than for non-infecting serotypes or related orthoflaviviruses, supporting the hypothesis that anti-EDIII antibodies provide greater specificity (Fig 4). SNAP-tagged EDIII antigens displayed similar patterns to untagged EDIII but with potential for enhanced sensitivity and specificity. Unlike non-SNAP-tagged antigens, which bind covalently to beads without controlled orientation and may result in heterogeneous presentation and partial masking of epitopes, SNAP-tagged antigens are site-specifically coupled via the SNAP domain using the BG-PEG-NH_2_ substrate. This approach leaves the viral antigen fully exposed for antibody recognition and may reduce nonspecific binding and background cross-reactivity, thereby improving assay performance. However, larger validation studies will be required to quantitatively confirm these potential advantages.

Responses to JEV provided further evidence of orthoflaviviruses cross-reactivity. In Senegal, where no JEV circulation has been reported, strong IgG responses against E and NS1 were nonetheless detected, suggesting cross-reactivity with other orthoflaviviruses. In Bangladesh, where JEV is known to circulate [21], strong IgG responses against E and NS1 were also observed. However, the lack of corresponding responses to SNAP-tagged EDIII indicates that much of this reactivity may reflect cross-reactivity with other orthoflaviviruses rather than JEV-specific infection. For both USUV and WNV, interpretation of IgG responses in non-endemic regions was complicated by cross-reactivity. Although no USUV circulation has been documented in Bangladesh or the Philippines, high NS1-specific IgG levels were observed. Similarly, WNV-specific responses were elevated in all sites despite confirmed circulation only in Senegal [42]. In both cases, SNAP-tagged EDIII antigens elicited low IgG signals in non-endemic regions, with a bimodal distribution in Senegal suggestive of true WNV exposure. These findings support the use of engineered envelope antigens for improved specificity.

ZIKV circulation has been documented in both Africa and Asia, but with notable regional differences. In Senegal where ZIKV circulation has been well documented [41] and in Bangladesh, where circulation appears limited, with a single confirmed case reported in 2016 [43] and a cluster of five cases identified in 2023 [44], strong IgG responses against VLP, U.NS1, and Su.NS1 antigens were observed. In contrast, responses to EDIII and SNAP-tagged EDIII antigens were detected exclusively in Senegalese samples, consistent with true ZIKV exposure in this setting and supporting the interpretation that reactivity observed in Bangladesh may not reflect true ZIKV circulation. Similarly, YFV circulation has been well documented in West Africa, including Senegal, where serological surveys, vector studies, and recurrent outbreaks confirm endemic transmission [45] and also vaccination campaigns have occurred, whereas in Bangladesh no evidence of YFV circulation has been reported. Indeed, we observed high IgG responses against NS1 and E antigens in samples from both regions. However, responses to SNAP-tagged EDIII antigen were detected only in Senegalese samples, consistent with true YFV exposure or vaccination responses. At present, our in-house multiplex bead-based immunoassay does not allow us to discriminate between antibody responses induced by natural YFV infection and those generated by vaccination.

IgG responses following DENV1 infection (Fig 4A) were less pronounced than those observed for CHIKV, likely due to broader cross-reactivity among orthoflaviviruses. In the Philippines, co-circulation [46] of DENV, ZIKV [47], and JEV [48] complicates clinical diagnosis and public health responses.

In Fig 4B, increases in IgG levels against multiple orthoflaviviruses antigens indicate that DENV1 EDIII and SNAP-tagged EDIII provide improved discrimination between orthoflaviviruses infections. In contrast, NS1 and VLPs exhibited higher cross-reactivity. Although IgG-based assays are generally affected by cross-reactivity due to affinity maturation and class switching, a similar pattern was observed for both IgM and IgA responses (Figs C, D, F, G, J, K, O, P, T, U X and Y in S1 Appendix). This likely reflects the high degree of structural similarity among orthoflaviviruses antigens, particularly in conserved regions of NS1 and the envelope protein. Early polyclonal B cell activation, especially during secondary or sequential orthoflaviviruses infections, may also contribute to cross-reactive IgM and IgA production [49]. The consistently elevated cross-reactivity observed with VLPs antigens across all isotypes highlights the role of epitope conservation in shaping the humoral immune response.

To investigate the association between serological profiles and functional immunity, a two-step Random Forest approach to predict PRNT-based neutralizing antibody responses using serological data was applied. Our findings underscore that the strength of serology as a surrogate for neutralization depends heavily on the virus and background levels of cross-reactive immunity. For CHIKV, a strong correlation between IgG responses, particularly to E2 and VLP antigens, and PRNT titers were observed, confirming that our in-house serological assay reliably reflects functional antibody activity. Both classification and regression models performed robustly, accurately identifying seropositive individuals and quantitatively predicting PRNT titers. These results support that the multiplex immunoassay data may serve as a reliable surrogate for PRNT in CHIKV serodiagnostics, enabling both classification and quantitative prediction of neutralizing capacity.

In contrast, predictive performance was substantially lower for DENV1, where IgG-based models struggled to accurately estimate neutralization. This disparity likely reflects the high degree of cross-reactivity and complex patterns of immunodominance and original antigenic sin among co-circulating orthoflaviviruses, which disrupts the relationship between binding and neutralizing antibodies. Consequently, IgG binding measurements appear to be less reliable proxies of neutralizing activity for DENV compared with CHIKV. This represents an important limitation for the use of multiplex serology to infer functional immunity against orthoflaviviruses. These results highlight that while machine learning tools can enhance the utility of multiplex serology, their predictive power depends on a clear immunological signal. Going forward, integrating serology with machine learning holds promise for scalable immunosurveillance, provided antigen selection and immunological context are carefully considered.

Our findings highlight the widespread issue of cross-reactivity among alphaviruses and orthoflaviviruses in serological assays and the resulting challenges for accurate serosurveillance. The use of SNAP-tagged EDIII and E2 antigens demonstrated improved specificity across multiple virus targets, emphasizing the importance of antigen engineering and the strategic use of multiple antigens in the development of reliable serosurveillance tools. Regional differences in IgG profiles further underscore the need for context-specific validation of serological markers, especially in areas with overlapping arbovirus transmission. Our longitudinal approach, in contrast to cross-sectional data (Fig 1), reveals the temporal dynamics of IgG responses and strengthens the ability to distinguish true infection from background cross-reactivity. Our study has several limitations that should be acknowledged. Most notably, the number of samples from individual with PCR-confirmed arbovirus infection was limited, and we did not have access to commercially available reference samples or larger number of samples with PCR-confirmed arboviruses infections, which limited our ability to fully validate the assay and include standardized positive and negative controls. Despite these limits, our results provide valuable insights into arboviruses serology across regions and highlight the importance of future studies incorporating larger, well-defined cohorts and reference panels.

## Supporting information

S1 Appendix Table A Detailed description of the deep-well plate layout. **Measuring IgG avidity with multiplex immunoassayFig A Spearman correlations between Tetracore MIA and in-house MIA recombinant NS1 antigens across orthoflaviviruses.** Scatterplots showing correlations between MFI values measured with Tetracore MIA and in-house MIA recombinant NS1 antigens across the same virus. Each point represents a sample diluted for the tetracore assay at 1/500 and at 1/400 with the in-house MIA. Blue lines show the linear regression fit, and the R² value represents the squared Spearman correlation coefficient for the pairwise comparison.**Fig B Pairwise Correlation of Antigen-Specific Antibody Responses Across Multiple Viruses across four countries.** Heatmap showing the pairwise Pearson correlation coefficients between antigen-specific antibody responses measured across multiple viral antigens. Each cell represents the correlation between two antigens, with red indicating positive correlation and blue indicating negative correlation (scale bar: -1 to 1).**Fig C Alphaviruses IgA antibody kinetics in individuals with RT-PCR-confirmed CHIKV infection. (A)** Longitudinal IgA antibody responses to CHIKV and other Alphaviruses in 4/12 representative infected individuals. Each column represents an infected individual, with the upper row showing responses to CHIKV-specific antigens (NSP, E2, VLP, and SNAP + E2) and the lower row displaying responses to other Alphaviruses antigens. The dashed vertical line indicates the date of RT-PCR-confirmed CHIKV infection. Antigens represented with dashed lines correspond to SNAP antigens. **(B)** Alphaviruses antibody boost following RT-PCR-confirmed CHIKV infection. The ratio between nMFI IgA between day 21 and day 0 after RT-PCR-confirmed CHIKV infection is presented. The dashed dotted line represents a 4-fold increase in nMFI. Each antigen is represented by a unique colour, defined in panel B.**Fig D Alphaviruses IgM antibody kinetics in individuals with RT-PCR-confirmed CHIKV infection. (A)** Longitudinal IgM antibody responses to CHIKV and other Alphaviruses in 4/12 representative infected individuals. Each column represents an infected individual, with the upper row showing responses to CHIKV-specific antigens (NSP, E2, VLP, and SNAP + E2) and the lower row displaying responses to other Alphaviruses antigens. The dashed vertical line indicates the date of RT-PCR-confirmed CHIKV infection. Antigens represented with dashed lines correspond to SNAP antigens. **(B)** Alphaviruses antibody boost following RT-PCR-confirmed CHIKV infection. The ratio between nMFI IgM between day 21 and day 0 after RT-PCR-confirmed CHIKV infection is presented. The dashed dotted line represents a 4-fold increase in nMFI. Each antigen is represented by a unique colour, defined in panel B.**Fig E Alphaviruses IgG avidity index kinetics in individuals with RT-PCR-confirmed CHIKV infection.** Longitudinal IgG avidity index to CHIKV and other Alphaviruses in 4/10 representative infected individuals. Each column represents an infected individual, with the upper row showing responses to CHIKV-specific antigens (NSP, E2, VLP, and SNAP + E2) and the lower row displaying responses to other Alphaviruses antigens. The dashed vertical line indicates the date of RT-PCR-confirmed CHIKV infection. Antigens represented with dashed lines correspond to SNAP antigens.**Fig F Orthoflaviviruses IgA antibody kinetics in individuals with RT-PCR-confirmed DENV1 infection. (A)** Longitudinal IgA antibody responses to DENV1 and other orthoflaviviruses in 4/10 infected individuals. Each column represents an infected individual, with the upper row showing responses to DENV-1-specific antigens (VLP, NS1, EDIII and EDIII + SNAP) and the lower rows displaying responses to other orthoflaviviruses antigens. The dashed vertical line indicates the date of RT-PCR-confirmed DENV-1 infection. Antigens represented with dashed lines correspond to SNAP antigens. **(B)** Orthoflaviviruses antibody boost following RT-PCR-confirmed DENV-1 infection. The ratio between nMFI IgA between day 21 and day 0 after RT-PCR-confirmed DENV-1 infection is presented. The dashed dotted line represents a 4-fold increase in nMFI. Each antigen is represented by a unique colour, defined in panel B.**Fig G Orthoflaviviruses IgM antibody kinetics in individuals with RT-PCR-confirmed DENV-1 infection. (A)** Longitudinal IgM antibody responses to DENV-1 and other orthoflaviviruses in 4/10 infected individuals. Each column represents an infected individual, with the upper row showing responses to DENV-1-specific antigens (VLP, NS1, EDIII and EDIII + SNAP) and the lower rows displaying responses to other orthoflaviviruses antigens. The dashed vertical line indicates the date of RT-PCR-confirmed DENV-1 infection. Antigens represented with dashed lines correspond to SNAP antigens. **(B)** Orthoflaviviruses antibody boost following RT-PCR-confirmed DENV-1 infection. The ratio between nMFI IgM between day 21 and day 0 after RT-PCR-confirmed DENV-1 infection is presented. The dashed dotted line represents a 4-fold increase in nMFI. Each antigen is represented by a unique colour, defined in panel B.**Fig H Orthoflaviviruses IgG avidity index kinetics in individuals with RT-PCR-confirmed DENV1 infection.** Longitudinal IgG avidity index to DENV1 and other orthoflaviviruses in 4/12 representative infected individuals. Each column represents an infected individual, with the upper row showing responses to DENV-1-specific antigens (VLP, NS1, EDIII and EDIII + SNAP) and the lower rows displaying responses to other orthoflaviviruses antigens. The dashed vertical line indicates the date of RT-PCR-confirmed DENV1 infection. Antigens represented with dashed lines correspond to SNAP antigens. Antigens represented with dashed lines correspond to SNAP antigens.**Fig I Orthoflaviviruses IgG antibody kinetics in individuals with RT-PCR-confirmed DENV2 infection. (A)** Longitudinal IgG antibody responses to DENV-2 and other orthoflaviviruses in 4/6 representative infected individuals. Each column represents an infected individual, with the upper row showing responses to DENV-2-specific antigens (VLP, NS1, EDIII and EDIII + SNAP) and the lower rows displaying responses to other orthoflaviviruses antigens. The dashed vertical line indicates the date of RT-PCR-confirmed DENV-2 infection. Antigens represented with dashed lines correspond to SNAP antigens. **(B)** Orthoflaviviruses antibody boost following RT-PCR-confirmed DENV-2 infection. The ratio between nMFI IgG between day 21 and day 0 after RT-PCR-confirmed DENV2 infection is presented. The dashed dotted line represents a 4-fold increase in nMFI. Each antigen is represented by a unique colour, defined in panel B.**Fig J Orthoflaviviruses IgA antibody kinetics in individuals with RT-PCR-confirmed DENV-2 infection. (A)** Longitudinal IgA antibody responses to DENV-2 and other orthoflaviviruses in 4/6 representative infected individuals. Each column represents an infected individual, with the upper row showing responses to DENV-2-specific antigens (VLP, NS1, EDIII and EDIII + SNAP) and the lower rows displaying responses to other orthoflaviviruses antigens. The dashed vertical line indicates the date of RT-PCR-confirmed DENV-2 infection. Antigens represented with dashed lines correspond to SNAP antigens. **(B)** Orthoflaviviruses antibody boost following RT-PCR-confirmed DENV-2 infection. The ratio between nMFI IgA between day 21 and day 0 after RT-PCR-confirmed DENV-2 infection is presented. The dashed dotted line represents a 4-fold increase in nMFI. Each antigen is represented by a unique colour, defined in panel B.**Fig K Orthoflaviviruses IgM antibody kinetics in individuals with RT-PCR-confirmed DENV-2 infection. (A)** Longitudinal IgM antibody responses to DENV-2 and other orthoflaviviruses in 4/6 representative infected individuals. Each column represents an infected individual, with the upper row showing responses to DENV-2-specific antigens (VLP, NS1, EDIII and EDIII + SNAP) and the lower rows displaying responses to other orthoflaviviruses antigens. The dashed vertical line indicates the date of RT-PCR-confirmed DENV-2 infection. Antigens represented with dashed lines correspond to SNAP antigens. **(B)** Orthoflaviviruses antibody boost following RT-PCR-confirmed DENV-2 infection. The ratio between nMFI IgM between day 21 and day 0 after RT-PCR-confirmed DENV-2 infection is presented. The dashed dotted line represents a 4-fold increase in nMFI. Each antigen is represented by a unique colour, defined in panel B.**Fig L Orthoflaviviruses IgG avidity index kinetics in individuals with RT-PCR-confirmed DENV-2 infection.** Longitudinal IgG avidity index to DENV-2 and other orthoflaviviruses in 4/6 representative infected individuals. Each column represents an infected individual, with the upper row showing responses to DENV-2-specific antigens (VLP, NS1, EDIII and EDIII + SNAP) and the lower rows displaying responses to other orthoflaviviruses antigens. The dashed vertical line indicates the date of RT-PCR-confirmed DENV-2 infection. Antigens represented with dashed lines correspond to SNAP antigens. **Fig M Random Forest algorithms to predict DENV-2 PRNT.** Comparison of DENV-2 PRNT and IgG levels for **(A)** DENV-2 NS1 (nMFI) and **(B)** DENV-2 VLP (nMFI). **(C)** Classification of DENV2 PRNT positivity using Random Forest step one. The boxplots represent the distribution of importance scores for the tested antigens. Features marked in green (confirmed) were identified as significantly contributing to the classification, while red (rejected) features did not provide meaningful classification power. The yellow (shadow features) serve as controls to determine the threshold for importance. **(D)** Prediction probability of DENV-2 PRNT positivity. PRNT positive samples are shown in blue and PRNT negative samples are shown in red. **(E)** Regression of DENV-2 PRNT using Random Forest step two. **(F)** Aggregated predictions from the out-of-bag step in the second Random Forest model for PRNT titer regression. This figure compares the actual (observed) PRNT titers with the model-predicted PRNT values obtained from the Random Forest regression. Each point represents a sample, with colors indicating the predicted probability of PRNT positivity (ranging from purple [low probability] to yellow [high probability]). The dashed diagonal line represents perfect agreement between observed and predicted PRNT values. Points clustering along this line indicate accurate predictions, while deviations suggest potential model limitations or outliers.**Fig N Orthoflaviviruses IgG antibody kinetics in individuals with RT-PCR-confirmed DENV-3 infection. (A)** Longitudinal IgG antibody responses to DENV-3 and other Orthoflaviviruses in 2/2 infected individuals. Each column represents an infected individual, with the upper row showing responses to DENV-3-specific antigens (VLP, NS1, EDIII and EDIII + SNAP) and the lower rows displaying responses to other Orthoflaviviruses antigens. The dashed vertical line indicates the date of RT-PCR-confirmed DENV-3 infection. Antigens represented with dashed lines correspond to SNAP antigens. **(B)** Flavivirus antibody boost following RT-PCR-confirmed DENV-3 infection. The ratio between nMFI IgG between day 21 and day 0 after RT-PCR-confirmed DENV-3 infection is presented. The dashed dotted line represents a 4-fold increase in nMFI. Each antigen is represented by a unique colour, defined in panel B.**Fig O Orthoflaviviruses IgA antibody kinetics in individuals with RT-PCR-confirmed DENV-3 infection. (A)** Longitudinal IgA antibody responses to DENV-3 and other Orthoflaviviruses in 2/2 infected individuals. Each column represents an infected individual, with the upper row showing responses to DENV-3-specific antigens (VLP, NS1, EDIII and EDIII + SNAP) and the lower rows displaying responses to other Orthoflaviviruses antigens. The dashed vertical line indicates the date of RT-PCR-confirmed DENV-3 infection. Antigens represented with dashed lines correspond to SNAP antigens. **(B)** Orthoflaviviruses antibody boost following RT-PCR-confirmed DENV-3 infection. The ratio between nMFI IgA between day 21 and day 0 after RT-PCR-confirmed DENV-3 infection is presented. The dashed dotted line represents a 4-fold increase in nMFI. Each antigen is represented by a unique colour, defined in panel B.**Fig P Orthoflaviviruses IgM antibody kinetics in individuals with RT-PCR-confirmed DENV-3 infection. (A)** Longitudinal IgM antibody responses to DENV-3 and other Orthoflaviviruses in 2/2 infected individuals. Each column represents an infected individual, with the upper row showing responses to DENV-3-specific antigens (VLP, NS1, EDIII and EDIII + SNAP) and the lower rows displaying responses to other Orthoflaviviruses antigens. The dashed vertical line indicates the date of RT-PCR-confirmed DENV-3 infection. Antigens represented with dashed lines correspond to SNAP antigens. **(B)** Orthoflaviviruses antibody boost following RT-PCR-confirmed DENV-3 infection. The ratio between nMFI IgM between day 21 and day 0 after RT-PCR-confirmed DENV-3 infection is presented. The dashed dotted line represents a 4-fold increase in nMFI. Each antigen is represented by a unique colour, defined in panel B.**Fig Q Orthoflaviviruses IgG avidity index kinetics in individuals with RT-PCR-confirmed DENV-3 infection.** Longitudinal IgG avidity index to DENV-3 and other Orthoflaviviruses in 2/2 infected individuals. Each column represents an infected individual, with the upper row showing responses to DENV-3-specific antigens (VLP, NS1, EDIII and EDIII + SNAP) and the lower rows displaying responses to other Orthoflaviviruses antigens. The dashed vertical line indicates the date of RT-PCR-confirmed DENV-3 infection. Antigens represented with dashed lines correspond to SNAP antigens. **Fig R Random Forest algorithms to predict DENV-3 PRNT.** Comparison of DENV-3 PRNT and IgG levels for **(A)** DENV-3 NS1 (nMFI) and **(B)** DENV-3 VLP (nMFI). **(C)** Classification of DENV-3 PRNT positivity using Random Forest step one. The boxplots represent the distribution of importance scores for the tested antigens. Features marked in green (confirmed) were identified as significantly contributing to the classification, while red (rejected) features did not provide meaningful classification power. The yellow (shadow features) serve as controls to determine the threshold for importance. **(D)** Prediction probability of DENV-3 PRNT positivity. PRNT positive samples are shown in blue and PRNT negative samples are shown in red. **(E)** Regression of DENV-3 PRNT using Random Forest step two. **(F)** Aggregated predictions from the out-of-bag step in the second Random Forest model for PRNT titer regression. This figure compares the actual (observed) PRNT titers with the model-predicted PRNT values obtained from the Random Forest regression. Each point represents a sample, with colors indicating the predicted probability of PRNT positivity (ranging from purple [low probability] to yellow [high probability]). The dashed diagonal line represents perfect agreement between observed and predicted PRNT values. Points clustering along this line indicate accurate predictions, while deviations suggest potential model limitations or outliers.**Fig S Orthoflaviviruses IgG antibody kinetics in individuals with RT-PCR-confirmed DENV-4 infection. (A)** Longitudinal IgG antibody responses to DENV-4 and other Orthoflaviviruses in 3/3 infected individuals. Each column represents an infected individual, with the upper row showing responses to DENV-4-specific antigens (VLP, NS1, EDIII and EDIII + SNAP) and the lower rows displaying responses to other Orthoflaviviruses antigens. The dashed vertical line indicates the date of RT-PCR-confirmed DENV-4 infection. Antigens represented with dashed lines correspond to SNAP antigens. Antigens represented with dashed lines correspond to SNAP antigens. **(B)** Flavivirus antibody boost following RT-PCR-confirmed DENV-4 infection. The ratio between nMFI IgG between day 21 and day 0 after RT-PCR-confirmed DENV-4 infection is presented. The dashed dotted line represents a 4-fold increase in nMFI. Each antigen is represented by a unique colour, defined in panel B.**Fig T Orthoflaviviruses IgA antibody kinetics in individuals with RT-PCR-confirmed DENV-4 infection. (A)** Longitudinal IgA antibody responses to DENV-4 and other Orthoflaviviruses in 3/3 infected individuals. Each column represents an infected individual, with the upper row showing responses to DENV-4-specific antigens (VLP, NS1, EDIII and EDIII + SNAP) and the lower rows displaying responses to other Orthoflaviviruses antigens. The dashed vertical line indicates the date of RT-PCR-confirmed DENV-4 infection. Antigens represented with dashed lines correspond to SNAP antigens. **(B)** Orthoflaviviruses antibody boost following RT-PCR-confirmed DENV-4 infection. The ratio between nMFI IgA between day 21 and day 0 after RT-PCR-confirmed DENV-4 infection is presented. The dashed dotted line represents a 4-fold increase in nMFI. Each antigen is represented by a unique colour, defined in panel B.**Fig U Orthoflaviviruses IgM antibody kinetics in individuals with RT-PCR-confirmed DENV-4 infection. (A)** Longitudinal IgM antibody responses to DENV-4 and other Orthoflaviviruses in 3/3 infected individuals. Each column represents an infected individual, with the upper row showing responses to DENV-4-specific antigens (VLP, NS1, EDIII and EDIII + SNAP) and the lower rows displaying responses to other Orthoflaviviruses antigens. The dashed vertical line indicates the date of RT-PCR-confirmed DENV-4 infection. Antigens represented with dashed lines correspond to SNAP antigens. **(B)** Orthoflaviviruses antibody boost following RT-PCR-confirmed DENV-4 infection. The ratio between nMFI IgM between day 21 and day 0 after RT-PCR-confirmed DENV-4 infection is presented. The dashed dotted line represents a 4-fold increase in nMFI. Each antigen is represented by a unique colour, defined in panel B.**Fig V Orthoflaviviruses IgG avidity index kinetics in individuals with RT-PCR-confirmed DENV-4 infection.** Longitudinal IgG avidity index to DENV-4 and other Orthoflaviviruses in 3/3 infected individuals. Each column represents an infected individual, with the upper row showing responses to DENV-4-specific antigens (VLP, NS1, EDIII and EDIII + SNAP) and the lower rows displaying responses to other Orthoflaviviruses antigens. The dashed vertical line indicates the date of RT-PCR-confirmed DENV-4 infection. Antigens represented with dashed lines correspond to SNAP antigens. **Fig W Orthoflaviviruses IgG antibody kinetics in individuals with RT-PCR-confirmed ZIKV infection. (A)** Longitudinal IgG antibody responses to ZIKV and other Orthoflaviviruses in 1/1 infected individual with the upper row showing responses to ZIKV-specific antigens (VLP, Su.NS1, U.NS1, EDIII and EDIII + SNAP) and the lower rows displaying responses to other Orthoflaviviruses antigens. The dashed vertical line indicates the date of RT-PCR-confirmed ZIKV infection. Antigens represented with dashed lines correspond to SNAP antigens. **(B)** Orthoflaviviruses antibody boost following RT-PCR-confirmed ZIKV infection. The ratio between nMFI IgG between day 21 and day 0 after RT-PCR-confirmed ZIKV infection is presented. The dashed dotted line represents a 4-fold increase in nMFI. Each antigen is represented by a unique colour, defined in panel B.**Fig X Orthoflaviviruses IgA antibody kinetics in individuals with RT-PCR-confirmed ZIKV infection. (A)** Longitudinal IgA antibody responses to ZIKV and other Orthoflaviviruses in 1/1 infected individual with the upper row showing responses to ZIKV-specific antigens (VLP, Su.NS1, U.NS1, EDIII and EDIII + SNAP) and the lower rows displaying responses to other Orthoflaviviruses antigens. The dashed vertical line indicates the date of RT-PCR-confirmed ZIKV infection. Antigens represented with dashed lines correspond to SNAP antigens. **(B)** Orthoflaviviruses antibody boost following RT-PCR-confirmed ZIKV infection. The ratio between nMFI IgA between day 21 and day 0 after RT-PCR-confirmed ZIKV infection is presented. The dashed dotted line represents a 4-fold increase in nMFI. Each antigen is represented by a unique colour, defined in panel B.**Fig Y Orthoflaviviruses IgM antibody kinetics in individuals with RT-PCR-confirmed ZIKV infection. (A)** Longitudinal IgM antibody responses to ZIKV and other Orthoflaviviruses in 1/1 infected individual with the upper row showing responses to ZIKV-specific antigens (VLP, Su.NS1, U.NS1, EDIII and EDIII + SNAP) and the lower rows displaying responses to other Orthoflaviviruses antigens. The dashed vertical line indicates the date of RT-PCR-confirmed ZIKV infection. Antigens represented with dashed lines correspond to SNAP antigens. **(B)** Orthoflaviviruses antibody boost following RT-PCR-confirmed ZIKV infection. The ratio between nMFI IgM between day 21 and day 0 after RT-PCR-confirmed ZIKV infection is presented. The dashed dotted line represents a 4-fold increase in nMFI. Each antigen is represented by a unique colour, defined in panel B.**Fig Z Orthoflaviviruses IgG avidity index kinetics in individuals with RT-PCR-confirmed ZIKV infection.** Longitudinal IgG avidity index to ZIKV and other Orthoflaviviruses in 1/1 infected individual with the upper row showing responses to ZIKV-specific antigens (VLP, NS1, EDIII and EDIII + SNAP) and the lower rows displaying responses to other Orthoflaviviruses antigens. The dashed vertical line indicates the date of RT-PCR-confirmed ZIKV infection. Antigens represented with dashed lines correspond to SNAP antigens.
(DOCX)

S2 Appendix Description of the Bindlt Software protocol (version 4.0.0.45) and details of the deep-well plates for automated coupling procedures. (PDF)

S1 Data IgG Antibody responses across populations. (XLSX)

S2 Data IgA and IgM Antibody responses across populations. (XLSX)

S3 Data IgG avidity Antibody responses across populations. (XLSX)
