# Supplementary material for: A multiplex serological assay for quantification of arbovirus antibody kinetics and neutralizing antibodies
Source: PLoS Negl Trop Dis. 2026 Sep 23;20(9):e0013704. doi: 10.1371/journal.pntd.0013704 (PMC13626472; doi:10.1371/journal.pntd.0013704)
Supplement: Supplementary file 1 [file pntd.0013704.s002.pdf]

General info

Protocol information

Protocol name	couplage_direct_200uL_well_A-D
Modified by	lagarcia
Kit name	-
Description	-

Sample layout

1	
2	
3	
4	
5	
6	
7	
8	
9	
10	
11	
12	

Reagent info

A (Peigne)		Couplage Part I	
**Name**	**Well volume [µl]**	**Total reagent volume [µl]**	**Type**
-	-	-	-

B (Beads)		Couplage Part I	
**Name**	**Well volume [µl]**	**Total reagent volume [µl]**	**Type**
Beads	208	-	Reagent

C (Wash 1)		Couplage Part I	
**Name**	**Well volume [µl]**	**Total reagent volume [µl]**	**Type**
Washing buffer	208	-	Reagent

D (Release START)		Couplage Part I	
**Name**	**Well volume [µl]**	**Total reagent volume [µl]**	**Type**
Activation Buffer / H2O	208	-	Reagent

E		Couplage Part I	
**Name**	**Well volume [µl]**	**Total reagent volume [µl]**	**Type**
-	-	-	-

F		Couplage Part I	
**Name**	**Well volume [µl]**	**Total reagent volume [µl]**	**Type**
-	-	-	-

G		Couplage Part I	
**Name**	**Well volume [µl]**	**Total reagent volume [µl]**	**Type**
-	-	-	-

H		Couplage Part I	
**Name**	**Well volume [µl]**	**Total reagent volume [µl]**	**Type**
-	-	-	-

A (Peigne)		Couplage Part II	
**Name**	**Well volume [µl]**	**Total reagent volume [µl]**	**Type**
-	-	-	-

B (Activation)		Couplage Part II	
**Name**	**Well volume [µl]**	**Total reagent volume [µl]**	**Type**
Activation Mix EDC + NHS	104	-	Reagent

C (Wash 1)		Couplage Part II	
**Name**	**Well volume [µl]**	**Total reagent volume [µl]**	**Type**
Coupling buffer	260	-	Reagent

D (Wash 2)		Couplage Part II	

Name	Well volume [µl]	Total reagent volume [µl]	Type
Coupling buffer	260	-	Reagent

**E (Coupling)**		**Couplage Part II**	
**Name**	**Well volume [µl]**	**Total reagent volume [µl]**	**Type**
Coupling buffer + proteins	1000	-	Reagent

**F (Wash 3)**		**Couplage Part II**	
**Name**	**Well volume [µl]**	**Total reagent volume [µl]**	**Type**
Wash/Storage Buffer	520	-	Reagent

**G (Wash 4)**		**Couplage Part II**	
**Name**	**Well volume [µl]**	**Total reagent volume [µl]**	**Type**
Wash 4	520	-	Reagent

**H (Release)**		**Couplage Part II**	
**Name**	**Well volume [µl]**	**Total reagent volume [µl]**	**Type**
Storage buffer	1000	-	Reagent

Dispensed reagents

The protocol does not contain dispensed reagents

Steps data

**Tip1**	**KingFisher Duo 12 tip comb**	
Pick-Up	Couplage Part I	(A) - Peigne
Beads	Couplage Part I	(B) - Beads
Beginning of step	Precollect	No
	Release beads	No
Mixing / heating:	Mixing time, speed	00:00:30, Medium
	Heating during mixing	No
End of step	Postmix	No
	Collect count	5
	Collect time [s]	30
	Post-temperature	No
Capture_beads	Couplage Part I	(B) - Beads
Beginning of step	Precollect	No
	Release beads	No
Mixing / heating:	Mixing time, speed	00:02:00, Slow
	Heating during mixing	No
End of step	Postmix	No
	Collect beads	No
	Post-temperature	No
Mix2	Couplage Part I	(C) - Wash 1
Beginning of step	Precollect	No
	Release time, speed	00:00:30, Medium
Mixing / heating:	Mixing time, speed	00:00:30, Medium
	Heating during mixing	No
End of step	Postmix	No
	Collect count	5
	Collect time [s]	30
	Post-temperature	No
ReleaseBeads1	Couplage Part I	(D) - Release START
	Release time, speed	00:00:30, Medium
Mix3	Couplage Part I	(D) - Release START
Beginning of step	Precollect	No
	Release beads	No
Mixing / heating:	Mixing time, speed	00:00:30, Medium
	Heating during mixing	No
End of step	Postmix	No
	Collect beads	No
	Post-temperature	No
Leave	Couplage Part II	(A) - Peigne
**Tip2**	**KingFisher Duo 12 tip comb**	

Pick-Up	Couplage Part II	(A) - Peigne

CollectBeads2	Couplage Part I	(D) - Release START
	Collect count	5
	Collect time [s]	30

Mix1	Couplage Part II	(B) - Activation
Beginning of step	Precollect	No
	Release time, speed	00:00:30, Medium
Mixing / heating:	Shake 1 time, speed	00:01:00, Medium
	Shake 2 time, speed	00:04:00, Paused
	Tip position when paused	Above well / tube surface
	Heating during mixing	No
End of step	Postmix	No
	Collect count	3
	Collect time [s]	30
	Post-temperature	No

Mix4	Couplage Part II	(C) - Wash 1
Beginning of step	Precollect	No
	Release time, speed	00:00:30, Medium
Mixing / heating:	Mixing time, speed	00:00:30, Medium
	Heating during mixing	No
End of step	Postmix	No
	Collect count	3
	Collect time [s]	30
	Post-temperature	No

Mix5	Couplage Part II	(D) - Wash 2
Beginning of step	Precollect	No
	Release time, speed	00:00:30, Medium
Mixing / heating:	Mixing time, speed	00:00:30, Medium
	Heating during mixing	No
End of step	Postmix	No
	Collect count	3
	Collect time [s]	30
	Post-temperature	No

Mix6	Couplage Part II	(E) - Coupling
Beginning of step	Precollect	No
	Release time, speed	00:00:30, Medium
Mixing / heating:	Shake 1 time, speed	00:01:00, Medium
	Shake 2 time, speed	00:04:30, Paused
	Loop count	21
	Tip position when paused	Tip edge in liquid
	Heating during mixing	No
End of step	Postmix	No
	Collect count	5
	Collect time [s]	30
	Post-temperature	No

Mix7	Couplage Part II	(F) - Wash 3
Beginning of step	Precollect	No
	Release time, speed	00:00:30, Medium
Mixing / heating:	Mixing time, speed	00:00:30, Medium
	Heating during mixing	No
End of step	Postmix	No
	Collect count	3
	Collect time [s]	30
	Post-temperature	No
Mix8	Couplage Part II	(G) - Wash 4
Beginning of step	Precollect	No
	Release time, speed	00:00:30, Medium
Mixing / heating:	Mixing time, speed	00:00:30, Medium
	Heating during mixing	No
End of step	Postmix	No
	Collect count	3
	Collect time [s]	30
	Post-temperature	No
ReleaseBeads2	Couplage Part II	(H) - Release
	Release time, speed	00:00:30, Medium
Leave	Couplage Part II	(A) - Peigne

Lot info

No lot numbers have been defined.
