# Supplementary material for: A multiplex serological assay for quantification of arbovirus antibody kinetics and neutralizing antibodies
Source: PLoS Negl Trop Dis. 2026 Sep 23;20(9):e0013704. doi: 10.1371/journal.pntd.0013704 (PMC13626472; doi:10.1371/journal.pntd.0013704)
Supplement: Supplementary file 2 [file pntd.0013704.s001.docx]

**S1 Appendix**

**Supplementary Methods**

***Automated coupling procedure***

**Deep-well plate 1**:

| **Row** | **Step** | **Buffer** |
| --- | --- | --- |
| A | Comb tip |  |
| B | 200 µL of stock uncoupled microspheres | Uncoupled microspheres+ Triton 0.05X |
| C | Wash 1 | 0.1M sodium phosphate (NaP) pH 6.2 + Triton 0.05X |
| D | Wash 1 | 0.1M sodium phosphate (NaP) pH 6.2 + Triton 0.05X |

**Deep-well plate 2**:

| **Row** | **Step** | **Buffer** |
| --- | --- | --- |
| A | Comb tip |  |
| B | Activation step | 0.1M sodium phosphate (NaP) pH 6.2, 10 mg/mL of EDC and 10 mg/mL sulfo-NHS + Triton 0.05X |
| C | Wash 2 | PBS 1X + Triton 0.05X |
| D | Wash 2 | PBS 1X + Triton 0.05X |
| E | Coupling step | 5-10 µg of recombinant proteins in PBS 1X + Triton 0.05X |
| F | Wash 3 | PBS1X + Triton 0.05X |
| G | Wash 3 | PBS-TBN (phosphate buffer saline supplemented with 1% bovine serum albumin, 0.02% sodium acid and 0.05% Tween-20) + Triton 0.05X |
| H | Storage step | PBS-TBN + Triton 0.05X |

Table A: Detailed description of the deep-well plate layout.

***Measuring IgG avidity with multiplex immunoassay***

For IgG avidity analysis, after reading the IgG plates on the Intelliflex® system, the plates were washed three times and resuspended in 100 µL of 8 M urea for 1 hour to dissociate low-avidity antibodies. Following this incubation, the plates were washed three times and incubated with a secondary antibody conjugated to R-phycoerythrin (2 µg/mL) specific for IgG for 15 minutes. The plates were then washed three times and resuspended in 150 µL of PBS 1X containing 0.05% Tween-20. Plates were read again using the Intelliflex® system at a low PMT setting, and the MFI was measured. This protocol was adapted from Garcia et al^21^.

***Fig A Spearman correlations between Tetracore MIA and in-house MIA recombinant NS1 antigens across Orthoflaviviruses.*** *Scatterplots showing correlations between MFI values measured with Tetracore MIA and in-house MIA recombinant NS1 antigens across the same virus. Each point represents a sample diluted for the tetracore assay at 1/500 and at 1/400 with the in-house MIA. Blue lines show the linear regression fit, and the R² value represents the squared Spearman correlation coefficient for the pairwise comparison.*

***Fig B Pairwise Correlation of Antigen-Specific Antibody Responses Across Multiple Viruses across four countries.*** *Heatmap showing the pairwise Pearson correlation coefficients between antigen-specific antibody responses measured across multiple viral antigens. Each cell represents the correlation between two antigens, with red indicating positive correlation and blue indicating negative correlation (scale bar: -1 to 1).*

***Fig C Alphaviruses IgA antibody kinetics in individuals with RT-PCR-confirmed CHIKV infection.*** **(*A)*** *Longitudinal IgA antibody responses to CHIKV and other Alphaviruses in 4/12 representative infected individuals*. *Each column represents an infected individual, with the upper row showing responses to CHIKV-specific antigens (NSP, E2, VLP, and SNAP + E2) and the lower row displaying responses to other Alphaviruses antigens. The dashed vertical line indicates the date of RT-PCR-confirmed CHIKV infection. Antigens represented with dashed lines correspond to SNAP antigens.* ***(B)*** *Alphaviruses antibody boost following RT-PCR-confirmed CHIKV infection. The ratio between nMFI IgA between day 21 and day 0 after RT-PCR-confirmed CHIKV infection is presented. The dashed dotted line represents a 4-fold increase in nMFI. Each antigen is represented by a unique colour, defined in panel B.*

***Fig D Alphaviruses IgM antibody kinetics in individuals with RT-PCR-confirmed CHIKV infection.*** **(*A)*** *Longitudinal IgM antibody responses to CHIKV and other Alphaviruses in 4/12 representative infected individuals*. *Each column represents an infected individual, with the upper row showing responses to CHIKV-specific antigens (NSP, E2, VLP, and SNAP + E2) and the lower row displaying responses to other Alphaviruses antigens. The dashed vertical line indicates the date of RT-PCR-confirmed CHIKV infection. Antigens represented with dashed lines correspond to SNAP antigens.* ***(B)*** *Alphaviruses antibody boost following RT-PCR-confirmed CHIKV infection. The ratio between nMFI IgM between day 21 and day 0 after RT-PCR-confirmed CHIKV infection is presented. The dashed dotted line represents a 4-fold increase in nMFI. Each antigen is represented by a unique colour, defined in panel B.*

***Fig E Alphaviruses IgG avidity index kinetics in individuals with RT-PCR-confirmed CHIKV infection.*** *Longitudinal IgG avidity index to CHIKV and other Alphaviruses in 4/10 representative infected individuals*. *Each column represents an infected individual, with the upper row showing responses to CHIKV-specific antigens (NSP, E2, VLP, and SNAP + E2) and the lower row displaying responses to other Alphaviruses antigens. The dashed vertical line indicates the date of RT-PCR-confirmed CHIKV infection. Antigens represented with dashed lines correspond to SNAP antigens.*

***Fig F Orthoflaviviruses IgA antibody kinetics in individuals with RT-PCR-confirmed DENV1 infection.*** **(*A)*** *Longitudinal IgA antibody responses to DENV1 and other orthoflaviviruses in 4/10 infected individuals*. *Each column represents an infected individual, with the upper row showing responses to DENV-1-specific antigens (VLP, NS1, EDIII and EDIII + SNAP) and the lower rows displaying responses to other orthoflaviviruses antigens. The dashed vertical line indicates the date of RT-PCR-confirmed DENV-1 infection. Antigens represented with dashed lines correspond to SNAP antigens.* ***(B)*** *Orthoflaviviruses antibody boost following RT-PCR-confirmed DENV-1 infection. The ratio between nMFI IgA between day 21 and day 0 after RT-PCR-confirmed DENV-1 infection is presented. The dashed dotted line represents a 4-fold increase in nMFI. Each antigen is represented by a unique colour, defined in panel B.*

***Fig G Orthoflaviviruses IgM antibody kinetics in individuals with RT-PCR-confirmed DENV-1 infection.*** **(*A)*** *Longitudinal IgM antibody responses to DENV-1 and other orthoflaviviruses in 4/10 infected individuals*. *Each column represents an infected individual, with the upper row showing responses to DENV-1-specific antigens (VLP, NS1, EDIII and EDIII + SNAP) and the lower rows displaying responses to other orthoflaviviruses antigens. The dashed vertical line indicates the date of RT-PCR-confirmed DENV-1 infection. Antigens represented with dashed lines correspond to SNAP antigens.* ***(B)*** *Orthoflaviviruses antibody boost following RT-PCR-confirmed DENV-1 infection. The ratio between nMFI IgM between day 21 and day 0 after RT-PCR-confirmed DENV-1 infection is presented. The dashed dotted line represents a 4-fold increase in nMFI. Each antigen is represented by a unique colour, defined in panel B.*

***Fig H Orthoflaviviruses IgG avidity index kinetics in individuals with RT-PCR-confirmed DENV1 infection.*** *Longitudinal IgG avidity index to DENV1 and other orthoflaviviruses in 4/12 representative infected individuals*. *Each column represents an infected individual, with the upper row showing responses to DENV-1-specific antigens (VLP, NS1, EDIII and EDIII + SNAP) and the lower rows displaying responses to other orthoflaviviruses antigens. The dashed vertical line indicates the date of RT-PCR-confirmed DENV1 infection. Antigens represented with dashed lines correspond to SNAP antigens. Antigens represented with dashed lines correspond to SNAP antigens.*

***Fig I Orthoflaviviruses IgG antibody kinetics in individuals with RT-PCR-confirmed DENV2 infection.*** **(*A)*** *Longitudinal IgG antibody responses to DENV-2 and other orthoflaviviruses in 4/6 representative infected individuals*. *Each column represents an infected individual, with the upper row showing responses to DENV-2-specific antigens (VLP, NS1, EDIII and EDIII + SNAP) and the lower rows displaying responses to other orthoflaviviruses antigens. The dashed vertical line indicates the date of RT-PCR-confirmed DENV-2 infection. Antigens represented with dashed lines correspond to SNAP antigens.* ***(B)*** *Orthoflaviviruses antibody boost following RT-PCR-confirmed DENV-2 infection. The ratio between nMFI IgG between day 21 and day 0 after RT-PCR-confirmed DENV2 infection is presented. The dashed dotted line represents a 4-fold increase in nMFI. Each antigen is represented by a unique colour, defined in panel B.*

***Fig J Orthoflaviviruses IgA antibody kinetics in individuals with RT-PCR-confirmed DENV-2 infection.*** **(*A)*** *Longitudinal IgA antibody responses to DENV-2 and other orthoflaviviruses in 4/6 representative infected individuals*. *Each column represents an infected individual, with the upper row showing responses to DENV-2-specific antigens (VLP, NS1, EDIII and EDIII + SNAP) and the lower rows displaying responses to other orthoflaviviruses antigens. The dashed vertical line indicates the date of RT-PCR-confirmed DENV-2 infection. Antigens represented with dashed lines correspond to SNAP antigens.* ***(B)*** *Orthoflaviviruses antibody boost following RT-PCR-confirmed DENV-2 infection. The ratio between nMFI IgA between day 21 and day 0 after RT-PCR-confirmed DENV-2 infection is presented. The dashed dotted line represents a 4-fold increase in nMFI. Each antigen is represented by a unique colour, defined in panel B.*

***Fig K Orthoflaviviruses IgM antibody kinetics in individuals with RT-PCR-confirmed DENV-2 infection.*** **(*A)*** *Longitudinal IgM antibody responses to DENV-2 and other orthoflaviviruses in 4/6 representative infected individuals*. *Each column represents an infected individual, with the upper row showing responses to DENV-2-specific antigens (VLP, NS1, EDIII and EDIII + SNAP) and the lower rows displaying responses to other orthoflaviviruses antigens. The dashed vertical line indicates the date of RT-PCR-confirmed DENV-2 infection. Antigens represented with dashed lines correspond to SNAP antigens.* ***(B)*** *Orthoflaviviruses antibody boost following RT-PCR-confirmed DENV-2 infection. The ratio between nMFI IgM between day 21 and day 0 after RT-PCR-confirmed DENV-2 infection is presented. The dashed dotted line represents a 4-fold increase in nMFI. Each antigen is represented by a unique colour, defined in panel B.*

***Fig L Orthoflaviviruses IgG avidity index kinetics in individuals with RT-PCR-confirmed DENV-2 infection.*** *Longitudinal IgG avidity index to DENV-2 and other orthoflaviviruses in 4/6 representative infected individuals*. *Each column represents an infected individual, with the upper row showing responses to DENV-2-specific antigens (VLP, NS1, EDIII and EDIII + SNAP) and the lower rows displaying responses to other orthoflaviviruses antigens. The dashed vertical line indicates the date of RT-PCR-confirmed DENV-2 infection. Antigens represented with dashed lines correspond to SNAP antigens.*

***Fig M Random Forest algorithms to predict DENV-2 PRNT.*** *Comparison of DENV-2 PRNT and IgG levels for* ***(A)*** *DENV-2 NS1 (nMFI) and* ***(B)*** *DENV-2 VLP (nMFI).* **(C*)*** *Classification of DENV2 PRNT positivity using Random Forest step one. The boxplots represent the distribution of importance scores for the tested antigens. Features marked in green (confirmed) were identified as significantly contributing to the classification, while red (rejected) features did not provide meaningful classification power. The yellow (shadow features) serve as controls to determine the threshold for importance.* **(*D)*** *Prediction probability of DENV-2 PRNT positivity. PRNT positive samples are shown in blue and PRNT negative samples are shown in red.* **(*E)*** *Regression of DENV-2 PRNT using Random Forest step two.* **(F*)*** *Aggregated predictions from the out-of-bag step in the second Random Forest model for PRNT titer regression. This figure compares the actual (observed) PRNT titers with the model-predicted PRNT values obtained from the Random Forest regression. Each point represents a sample, with colors indicating the predicted probability of PRNT positivity (ranging from purple [low probability] to yellow [high probability]). The dashed diagonal line represents perfect agreement between observed and predicted PRNT values. Points clustering along this line indicate accurate predictions, while deviations suggest potential model limitations or outliers.*

***Fig N Orthoflaviviruses IgG antibody kinetics in individuals with RT-PCR-confirmed DENV-3 infection.*** **(*A)*** *Longitudinal IgG antibody responses to DENV-3 and other Orthoflaviviruses in 2/2 infected individuals*. *Each column represents an infected individual, with the upper row showing responses to DENV-3-specific antigens (VLP, NS1, EDIII and EDIII + SNAP) and the lower rows displaying responses to other Orthoflaviviruses antigens. The dashed vertical line indicates the date of RT-PCR-confirmed DENV-3 infection. Antigens represented with dashed lines correspond to SNAP antigens.* ***(B)*** *Flavivirus antibody boost following RT-PCR-confirmed DENV-3 infection. The ratio between nMFI IgG between day 21 and day 0 after RT-PCR-confirmed DENV-3 infection is presented. The dashed dotted line represents a 4-fold increase in nMFI. Each antigen is represented by a unique colour, defined in panel B.*

***Fig O Orthoflaviviruses IgA antibody kinetics in individuals with RT-PCR-confirmed DENV-3 infection.*** **(*A)*** *Longitudinal IgA antibody responses to DENV-3 and other Orthoflaviviruses in 2/2 infected individuals*. *Each column represents an infected individual, with the upper row showing responses to DENV-3-specific antigens (VLP, NS1, EDIII and EDIII + SNAP) and the lower rows displaying responses to other Orthoflaviviruses antigens. The dashed vertical line indicates the date of RT-PCR-confirmed DENV-3 infection. Antigens represented with dashed lines correspond to SNAP antigens.* ***(B)*** *Orthoflaviviruses antibody boost following RT-PCR-confirmed DENV-3 infection. The ratio between nMFI IgA between day 21 and day 0 after RT-PCR-confirmed DENV-3 infection is presented. The dashed dotted line represents a 4-fold increase in nMFI. Each antigen is represented by a unique colour, defined in panel B.*

***Fig P Orthoflaviviruses IgM antibody kinetics in individuals with RT-PCR-confirmed DENV-3 infection.*** **(*A)*** *Longitudinal IgM antibody responses to DENV-3 and other Orthoflaviviruses in 2/2 infected individuals*. *Each column represents an infected individual, with the upper row showing responses to DENV-3-specific antigens (VLP, NS1, EDIII and EDIII + SNAP) and the lower rows displaying responses to other Orthoflaviviruses antigens. The dashed vertical line indicates the date of RT-PCR-confirmed DENV-3 infection. Antigens represented with dashed lines correspond to SNAP antigens.* ***(B)*** *Orthoflaviviruses antibody boost following RT-PCR-confirmed DENV-3 infection. The ratio between nMFI IgM between day 21 and day 0 after RT-PCR-confirmed DENV-3 infection is presented. The dashed dotted line represents a 4-fold increase in nMFI. Each antigen is represented by a unique colour, defined in panel B.*

***Fig Q Orthoflaviviruses IgG avidity index kinetics in individuals with RT-PCR-confirmed DENV-3 infection.*** *Longitudinal IgG avidity index to DENV-3 and other Orthoflaviviruses in 2/2 infected individuals*. *Each column represents an infected individual, with the upper row showing responses to DENV-3-specific antigens (VLP, NS1, EDIII and EDIII + SNAP) and the lower rows displaying responses to other Orthoflaviviruses antigens. The dashed vertical line indicates the date of RT-PCR-confirmed DENV-3 infection. Antigens represented with dashed lines correspond to SNAP antigens.*

***Fig R Random Forest algorithms to predict DENV-3 PRNT.*** *Comparison of DENV-3 PRNT and IgG levels for* ***(A)*** *DENV-3 NS1 (nMFI) and* ***(B)*** *DENV-3 VLP (nMFI).* **(C*)*** *Classification of DENV-3 PRNT positivity using Random Forest step one. The boxplots represent the distribution of importance scores for the tested antigens. Features marked in green (confirmed) were identified as significantly contributing to the classification, while red (rejected) features did not provide meaningful classification power. The yellow (shadow features) serve as controls to determine the threshold for importance.* **(*D)*** *Prediction probability of DENV-3 PRNT positivity. PRNT positive samples are shown in blue and PRNT negative samples are shown in red.* **(*E)*** *Regression of DENV-3 PRNT using Random Forest step two.* **(F*)*** *Aggregated predictions from the out-of-bag step in the second Random Forest model for PRNT titer regression. This figure compares the actual (observed) PRNT titers with the model-predicted PRNT values obtained from the Random Forest regression. Each point represents a sample, with colors indicating the predicted probability of PRNT positivity (ranging from purple [low probability] to yellow [high probability]). The dashed diagonal line represents perfect agreement between observed and predicted PRNT values. Points clustering along this line indicate accurate predictions, while deviations suggest potential model limitations or outliers.*

***Fig S Orthoflaviviruses IgG antibody kinetics in individuals with RT-PCR-confirmed DENV-4 infection.*** **(*A)*** *Longitudinal IgG antibody responses to DENV-4 and other Orthoflaviviruses in 3/3 infected individuals*. *Each column represents an infected individual, with the upper row showing responses to DENV-4-specific antigens (VLP, NS1, EDIII and EDIII + SNAP) and the lower rows displaying responses to other Orthoflaviviruses antigens. The dashed vertical line indicates the date of RT-PCR-confirmed DENV-4 infection. Antigens represented with dashed lines correspond to SNAP antigens. Antigens represented with dashed lines correspond to SNAP antigens.* ***(B)*** *Flavivirus antibody boost following RT-PCR-confirmed DENV-4 infection. The ratio between nMFI IgG between day 21 and day 0 after RT-PCR-confirmed DENV-4 infection is presented. The dashed dotted line represents a 4-fold increase in nMFI. Each antigen is represented by a unique colour, defined in panel B.*

***Fig T Orthoflaviviruses IgA antibody kinetics in individuals with RT-PCR-confirmed DENV-4 infection.*** **(*A)*** *Longitudinal IgA antibody responses to DENV-4 and other Orthoflaviviruses in 3/3 infected individuals*. *Each column represents an infected individual, with the upper row showing responses to DENV-4-specific antigens (VLP, NS1, EDIII and EDIII + SNAP) and the lower rows displaying responses to other Orthoflaviviruses antigens. The dashed vertical line indicates the date of RT-PCR-confirmed DENV-4 infection. Antigens represented with dashed lines correspond to SNAP antigens.* ***(B)*** *Orthoflaviviruses antibody boost following RT-PCR-confirmed DENV-4 infection. The ratio between nMFI IgA between day 21 and day 0 after RT-PCR-confirmed DENV-4 infection is presented. The dashed dotted line represents a 4-fold increase in nMFI. Each antigen is represented by a unique colour, defined in panel B.*

***Fig U Orthoflaviviruses IgM antibody kinetics in individuals with RT-PCR-confirmed DENV-4 infection.*** **(*A)*** *Longitudinal IgM antibody responses to DENV-4 and other Orthoflaviviruses in 3/3 infected individuals*. *Each column represents an infected individual, with the upper row showing responses to DENV-4-specific antigens (VLP, NS1, EDIII and EDIII + SNAP) and the lower rows displaying responses to other Orthoflaviviruses antigens. The dashed vertical line indicates the date of RT-PCR-confirmed DENV-4 infection. Antigens represented with dashed lines correspond to SNAP antigens.* ***(B)*** *Orthoflaviviruses antibody boost following RT-PCR-confirmed DENV-4 infection. The ratio between nMFI IgM between day 21 and day 0 after RT-PCR-confirmed DENV-4 infection is presented. The dashed dotted line represents a 4-fold increase in nMFI. Each antigen is represented by a unique colour, defined in panel B.*

***Fig V Orthoflaviviruses IgG avidity index kinetics in individuals with RT-PCR-confirmed DENV-4 infection.*** *Longitudinal IgG avidity index to DENV-4 and other Orthoflaviviruses in 3/3 infected individuals*. *Each column represents an infected individual, with the upper row showing responses to DENV-4-specific antigens (VLP, NS1, EDIII and EDIII + SNAP) and the lower rows displaying responses to other Orthoflaviviruses antigens. The dashed vertical line indicates the date of RT-PCR-confirmed DENV-4 infection. Antigens represented with dashed lines correspond to SNAP antigens.*

***Fig W Orthoflaviviruses IgG antibody kinetics in individuals with RT-PCR-confirmed ZIKV infection.*** **(*A)*** *Longitudinal IgG antibody responses to ZIKV and other Orthoflaviviruses in 1/1 infected individual* *with the upper row showing responses to ZIKV-specific antigens (VLP, Su.NS1, U.NS1, EDIII and EDIII + SNAP) and the lower rows displaying responses to other Orthoflaviviruses antigens. The dashed vertical line indicates the date of RT-PCR-confirmed ZIKV infection. Antigens represented with dashed lines correspond to SNAP antigens.* ***(B)*** *Orthoflaviviruses antibody boost following RT-PCR-confirmed ZIKV infection. The ratio between nMFI IgG between day 21 and day 0 after RT-PCR-confirmed ZIKV infection is presented. The dashed dotted line represents a 4-fold increase in nMFI. Each antigen is represented by a unique colour, defined in panel B.*

***Fig X Orthoflaviviruses IgA antibody kinetics in individuals with RT-PCR-confirmed ZIKV infection.*** **(*A)*** *Longitudinal IgA antibody responses to ZIKV and other Orthoflaviviruses in 1/1 infected individual* *with the upper row showing responses to ZIKV-specific antigens (VLP, Su.NS1, U.NS1, EDIII and EDIII + SNAP) and the lower rows displaying responses to other Orthoflaviviruses antigens. The dashed vertical line indicates the date of RT-PCR-confirmed ZIKV infection. Antigens represented with dashed lines correspond to SNAP antigens.* ***(B)*** *Orthoflaviviruses antibody boost following RT-PCR-confirmed ZIKV infection. The ratio between nMFI IgA between day 21 and day 0 after RT-PCR-confirmed ZIKV infection is presented. The dashed dotted line represents a 4-fold increase in nMFI. Each antigen is represented by a unique colour, defined in panel B.*

***Fig Y Orthoflaviviruses IgM antibody kinetics in individuals with RT-PCR-confirmed ZIKV infection.*** **(*A)*** *Longitudinal IgM antibody responses to ZIKV and other Orthoflaviviruses in 1/1 infected individual* *with the upper row showing responses to ZIKV-specific antigens (VLP, Su.NS1, U.NS1, EDIII and EDIII + SNAP) and the lower rows displaying responses to other Orthoflaviviruses antigens. The dashed vertical line indicates the date of RT-PCR-confirmed ZIKV infection. Antigens represented with dashed lines correspond to SNAP antigens.* ***(B)*** *Orthoflaviviruses antibody boost following RT-PCR-confirmed ZIKV infection. The ratio between nMFI IgM between day 21 and day 0 after RT-PCR-confirmed ZIKV infection is presented. The dashed dotted line represents a 4-fold increase in nMFI. Each antigen is represented by a unique colour, defined in panel B.*

***Fig Z Orthoflaviviruses IgG avidity index kinetics in individuals with RT-PCR-confirmed ZIKV infection.*** *Longitudinal IgG avidity index to ZIKV and other Orthoflaviviruses in 1/1 infected individual* *with the upper row showing responses to ZIKV-specific antigens (VLP, NS1, EDIII and EDIII + SNAP) and the lower rows displaying responses to other Orthoflaviviruses antigens. The dashed vertical line indicates the date of RT-PCR-confirmed ZIKV infection. Antigens represented with dashed lines correspond to SNAP antigens.*
