# Supplementary material for: A multiplex serological assay for quantification of arbovirus antibody kinetics and neutralizing antibodies
Source: PLoS Negl Trop Dis. 2026 Sep 23;20(9):e0013704. doi: 10.1371/journal.pntd.0013704 (PMC13626472; doi:10.1371/journal.pntd.0013704)
Supplement: Supplementary file 3 [file pntd.0013704.s007.docx]

**PLOS Neglected Tropical Diseases Technical Check requests for your submission PNTD-D-25-01903R1 - [EMID:9f13a294d8195672]**

1) Please ensure that the CRediT author contributions listed for every co-author are completed accurately and in full.

At this stage, the following Authors/Authors require contributions: Scott C. Weaver, and Jason Rosado. Please ensure that the full contributions of each author are acknowledged in the "Add/Edit/Remove Authors" section of our submission form.

The list of CRediT author contributions may be found here: [https://urldefense.com/v3/__https://journals.plos.org/plosntds/s/authorship*loc-author-contributions__;Iw!!JFdNOqOXpB6UZW0!u_bugwA9O4M0k_8PcsmIbVxrSwRy6J2IDM0AwYKUlrnxwJlay50EL9dQ9JTw1BliTg7fIxIejNo64XrIcLG1VlA$](https://urldefense.com/v3/__https:/journals.plos.org/plosntds/s/authorship*loc-author-contributions__;Iw!!JFdNOqOXpB6UZW0!u_bugwA9O4M0k_8PcsmIbVxrSwRy6J2IDM0AwYKUlrnxwJlay50EL9dQ9JTw1BliTg7fIxIejNo64XrIcLG1VlA$)

The contributions of Scott Weaver and Jason Rosado have now been completed.

2) Please ensure that the Author List in your manuscript file matches the Author List in the online submission form including initials and order, specifically Stephane Petres is missing from the Manuscript Please use either initials or full names in both the manuscript and online submission form.

The Author List in the online manuscript form has been updated so that all authors appear in the same order as the Author List in the manuscript file (Garcia_PNTD-D-25-01903_Manuscript_clean.docx). Notably, we have verified this for Stephane Petres.

3) Please ensure that the Author List in your manuscript file matches the Author List in the online submission form exactly. Authors should be listed in the same order, and the order of individual first and last author names must be identical in both locations.

The Author List in the online manuscript form has been updated so that all authors appear in the same order as the Author List in the manuscript file (Garcia_PNTD-D-25-01903_Manuscript_clean.docx).

4) Thank you for including an Ethics Statement for your study. Please include:

i) A statement that formal consent was obtained (must state whether verbal/written) OR the reason consent was not obtained (e.g. anonymity). NOTE: If child participants, the statement must declare that formal consent was obtained from the parent/guardian.]

The ethics statement has been updated as follows:

Informed written consent was obtained from all study participants. For children, informed written consent was provided by a parent/guardian.

5) Please amend your detailed Financial Disclosure statement. This is published with the article. It must therefore be completed in full sentences and contain the exact wording you wish to be published.

1) State the initials, alongside each funding source, of each author to receive each grant. For example: "This work was supported by the National Institutes of Health (####### to AM; ###### to CJ) and the National Science Foundation (###### to AM)."

2) State what role the funders took in the study. If the funders had no role in your study, please state: "The funders had no role in study design, data collection and analysis, decision to publish, or preparation of the manuscript.".

If you did not receive any funding for this study, please simply state: u201cThe authors received no specific funding for this work.u201d

The funding statement has been updated to denote which investigators received the funding.

The following statement has been included in the Funding statement:

The funders had no role in study design, data collection and analysis, decision to publish, or preparation of the manuscript.

6) Please ensure that the funders and grant numbers match between the Financial Disclosure field and the Funding Information tab in your submission form. Note that the funders must be provided in the same order in both places as well.

Currently, order of grants are different in both places.

We can confirm that the ordering of the funding and grant numbers is the same in both locations.

7) We have amended your Competing Interest statement to comply with journal style. We kindly ask that you double check the statement and let us know if anything is incorrect.

Yes, we agree with this statement.

8) We note that the author byline has changed since the last version of the manuscript.

This is correct. We are currently preparing the Authorship Change forms and will share them as soon as possible.

**Response required: PLOS Neglected Tropical Diseases Potential copyright issues for your submission PNTD-D-25-01903R1 - [EMID:44ae6408b36aa0c3]**

i) Figure Protocol_KingFisher_DuoPrime.pdf. Please confirm whether you drew the images / clip-art within the figure panels by hand. If you did not draw the images, please provide (a) a link to the source of the images or icons and their license / terms of use; or (b) written permission from the copyright holder to publish the images or icons under our CC BY 4.0 license. Alternatively, you may replace the images with open source alternatives. See these open source resources you may use to replace images / clip-art:

- https://commons.wikimedia.org

- <https://openclipart.org/>.

The file Protocol_KingFisher_DuoPrime.pdf contains information on a scientific protocol, and does not contain any images.
