# Supplementary material for: A multiplex serological assay for quantification of arbovirus antibody kinetics and neutralizing antibodies
Source: PLoS Negl Trop Dis. 2026 Sep 23;20(9):e0013704. doi: 10.1371/journal.pntd.0013704 (PMC13626472; doi:10.1371/journal.pntd.0013704)
Supplement: Supplementary file 4 [file pntd.0013704.s008.docx]

**Infectious Disease Epidemiology
& Analytics G5 Unit**

Institut Pasteur

25-28 Rue du Dr Roux

Paris 75015

France

**Dr Michael White**

michael.white@pasteur.fr

29^th^ May 2026

Dear Editor,

Related to our manuscript – “A Multiplex Serological Assay for Quantification of Arbovirus Antibody Kinetics and Neutralizing Antibodies” – please see below for a point by point response to the editorial requests detailed in your e-mail dated 29^th^ May 2026.

Yours sincerely,

Dr Michael White, on behalf of the authors

1) Please ensure that the affiliations of the author ( Jason Rosado) listed on the manuscript title page (CITY) do exactly match with the affiliations provided in the online submission form
NOTE: Affiliations should include a department (if applicable), an institution, a CITY, and a country

The affiliations of Jason Rosado and all other co-authors have been updated to ensure consistency with the manuscript.

2) Thank you for stating "Informed consent was obtained from all study participants. For children, informed consent was provided by a parent/guardian".

Please include a statement that formal consent was obtained (must state whether verbal/written) OR the reason consent was not obtained (e.g. anonymity). NOTE: If child participants, the statement must declare that formal consent  ((must state whether verbal/written) was obtained from the parent/guardian.

In the Ethics section, the text has been updated to explicitly state that written informed consent was obtained (lines 165 – 166).

3) Please upload all main figures as separate Figure files in .tif or .eps format. For more information about how to convert and format your figure files please see our guidelines: 
[https://urldefense.com/v3/__https://journals.plos.org/plosntds/s/figures__;!!JFdNOqOXpB6UZW0!uMAHZT9nJyRKpcL2bNWiDIMRargcRRQs5E6kUZXIV0vqNXUJqp75o2wa0XWrElgNpCJ90lB_Sygw8G5Anq2NC08$](https://urldefense.com/v3/__https:/journals.plos.org/plosntds/s/figures__;!!JFdNOqOXpB6UZW0!uMAHZT9nJyRKpcL2bNWiDIMRargcRRQs5E6kUZXIV0vqNXUJqp75o2wa0XWrElgNpCJ90lB_Sygw8G5Anq2NC08$)

All figures are now in .tif format.

 
4) Please amend your detailed Financial Disclosure statement. This is published with the article, therefore should be completed in full sentences and contain the exact wording you wish to be published.
i). State what role the funders took in the study. If the funders had no role in your study, please state: “The funders had no role in study design, data collection and analysis, decision to publish, or preparation of the manuscript.”
ii). If any authors received a salary from any of your funders, please state which authors and which funders.

This statement is provided in the Funding section of the manuscript (lines 629 – 630).

5) Please ensure that the funders and grant numbers match between the Financial Disclosure field and the Funding Information tab in your submission form. Note that the funders must be provided in the same order in both places as well.
Currently, order of grants are different in both places.

The statement provided in the Funding section of the manuscript is now identical to the statement provided in the Financial Disclosure field.

6) Please note that there should not be any identifying data in the Supporting Information file . If there is any such data, we ask that you please remove it. Prior to sharing human research participant data, authors should consult with an ethics committee to ensure data are shared in accordance with participant consent and all applicable local laws.

We can confirm that there is no identifying data in the Supporting Information file.
