# Supplementary material for: A multiplex serological assay for quantification of arbovirus antibody kinetics and neutralizing antibodies
Source: PLoS Negl Trop Dis. 2026 Sep 23;20(9):e0013704. doi: 10.1371/journal.pntd.0013704 (PMC13626472; doi:10.1371/journal.pntd.0013704)
Supplement: Supplementary file 5 [file pntd.0013704.s009.docx]

**Infectious Disease Epidemiology
& Analytics G5 Unit**

Institut Pasteur

25-28 Rue du Dr Roux

Paris 75015

France

**Dr Michael White**

michael.white@pasteur.fr

28^th^ July 2026

Dear Editor,

We would like to resubmit the revised version of our manuscript entitled “A Multiplex Serological Assay for Quantification of Arbovirus Antibody Kinetics and Neutralizing Antibodies” for further consideration for publication in *PLOS Neglected Tropical Diseases*.

We would like to sincerely thank the editors and reviewers for their careful evaluation of our manuscript and for their valuable comments and suggestions. We have carefully considered all remaining points raised during the review process and have revised the manuscript accordingly. In particular, we have updated two figures to address the reviewers’ comments.

We believe that these revisions have further improved the clarity and quality of the manuscript, and we hope that the revised version will now be suitable for publication in *PLOS Neglected Tropical Diseases*.

We look forward to your consideration of our revised manuscript.

Yours sincerely,

Dr Michael White, on behalf of the authors

**Reviewer #2:**

The objectives are clearly articulated. The authors explicitly state their goal: to develop, automate, and validate a high-throughput multiplex bead-based immunoassay to measure IgG, IgA, and IgM responses against a wide panel of 44 arbovirus antigens, and to explore antibody kinetics and predict plaque reduction neutralization test (PRNT) titers using machine learning. While framed more as a methodological validation than a rigid hypothesis-driven mechanistic paper, it clearly tests whether multiplex binding profiles can serve as a functional surrogate for virus-neutralizing activity. the study design is highly appropriate. It leverages a diverse panel of 647 samples stratified across four distinct geographic and epidemiologic regions: endemic settings (Senegal, Bangladesh, the Philippines) and a non-endemic control setting (France). It also cleverly incorporates both longitudinal cohorts with RT-PCR-confirmed infections to map kinetic boosts over time and cross-sectional panels with paired gold-standard neutralization data. the population cohorts are explicitly defined in the text and neatly organized in Table 1, providing details on expected exposure, sample size, median age, and sex distribution. Utilizing unexposed individuals from France alongside populations frequently exposed to co-circulating arboviruses provides the ideal physiological contrast needed to evaluate assay specificity and background cross-reactivity. The overall sample size ($n = 647$) is robust for mapping general antibody distributions and training the machine learning models. However, as the authors transparently acknowledge in the limitations, the subset of individuals with longitudinal, RT-PCR-confirmed infections is modest (e.g., 12 for CHIKV, 10 for DENV1, and single digits for other DENV serotypes). While sufficient for an exploratory descriptive study of kinetic trends, it limits broader statistical generalization of serotype-specific kinetics. The two-step Random Forest framework—separating classification (PRNT positive vs. negative) from regression (predicting log-titers)—coupled with Boruta feature selection is a statistically sound approach for handling high-dimensional multiplex array inputs. The use of out-of-bag (OOB) predictions provides a reliable form of internal cross-validation. there are no concerns. Institutional Review Board (IRB) approval details and explicit protocol registry numbers are documented for all four study sites. In response to prior editorial adjustments, the text now explicitly declares that written formal informed consent was obtained from all participants or their legal guardians.

We sincerely thank the reviewer for their thorough and positive assessment of our study. We appreciate the recognition that the study objectives, design, population selection, statistical methodology, and ethical considerations are clearly presented and appropriate for the aims of the work. We are also grateful that the reviewer acknowledged the transparency with which we discuss the limitations of the longitudinal cohorts and the improvements made to the manuscript regarding informed consent and ethical approvals.

The presented results track the structured objectives perfectly. The sequence flows logically from defining distribution profiles across countries (Figures 1 and 2), exploring longitudinal antibody boosts post-infection (Figures 3 and 4), to executing the machine learning neutralization modeling (Figures 5 and 6). the data is detailed thoroughly. The manuscript beautifully highlights a crucial biological contrast: binding antibodies translate to an excellent surrogate model for predicting functional neutralization in alphaviruses (CHIKV R2 = 0.71) but perform poorly for orthoflaviviruses (DENV1 R2 = 0.42) due to intricate cross-reactive profiles and immunodominance issues. The figures are exceptionally well-crafted, clear, and informative. The multi-panel alignment comparing actual vs. predicted titers alongside Boruta variable importance plots makes complex algorithmic outputs easily digestible at a glance. The authors have successfully corrected their formatting in accordance with journal specifications.

We sincerely thank the reviewer for this very positive evaluation of our Results section. We greatly appreciate the recognition that the analyses are logically organized, the data are clearly presented, and the figures effectively communicate the key findings. We are also pleased that the reviewer found the comparison between observed and predicted neutralization titers, as well as the Boruta variable importance analyses, to be informative and easy to interpret. Finally, we thank the reviewer for acknowledging the improvements made to the manuscript presentation and formatting.

the conclusions are well-supported. The data directly confirms that while a multiplex approach can vastly optimize high-throughput epidemiological surveillance, using total IgG binding metrics to infer functional immunity or specific neutralisation thresholds remains highly restricted for flaviviruses. The authors are candid about their biological and physical constraints. They discuss the small sample counts for PCR-confirmed reference cohorts, the lack of broader commercial reference standards, and the intrinsic inability of this platform to reliably distinguish between past natural yellow fever exposure and vaccination responses. The discussion carefully outlines how implementing site-specifically oriented, SNAP-tagged engineered envelope domains (EDIII and E2) can bypass the typical architectural pitfalls of random amine coupling. This advances serological method development by significantly lowering background noise and nonspecific cross-reactivity. Extensively. The authors emphasize the scalability, low sample volume demands, and cost-efficiency of this multiplex platform. They cleanly link the tool to its real-world implementation: mapping multi-pathogen transmission landscapes and shaping vaccine deployment strategies in areas plagued by co-circulating arboviruses.

We sincerely thank the reviewer for their positive evaluation of our conclusions and discussion. We greatly appreciate their recognition that the conclusions are appropriately supported by the data and that the manuscript transparently addresses the limitations of the study, including the limited number of PCR-confirmed reference samples, the lack of commercially available reference standards, and the challenges associated with distinguishing natural infection from vaccination. We are also pleased that the reviewer recognized the added value of the discussion regarding the rationale and potential benefits of site-specific coupling of SNAP-tagged antigens, as well as the translational relevance of the assay for large-scale serological surveillance and public health applications.

The manuscript is in excellent shape, reflecting rigorous responses to prior evaluation rounds. Only minor, optional formatting enhancements are suggested to polish readability:

Aesthetics in Figures 3 and 4: In Figure 3A and Figure 4A, some of the light gray line traces representing "other alphaviruses" or "other orthoflaviviruses" crowd the background of the individual facets. While acceptable, making these gray lines slightly more translucent would let the primary colored virus traces pop even more effectively.

We sincerely thank the reviewer for their very positive assessment of the revised manuscript. We appreciate this helpful suggestion to further improve figure readability. In response, we have increased the transparency of the light grey traces representing the other alphaviruses and orthoflaviviruses in Figures 3A and 4A.

Abbreviation Continuity: Check that all structural domains are consistently abbreviated following the authors' standardization exercise. Ensure that "structural polyprotein" (SP) or "non-structural protein" (NSP) are fully capitalized across all supplementary figure subtitles to match the main text layout.

We thank the reviewer for pointing out this potential inconsistency. We carefully checked all supplementary figure subtitles and confirmed that the terminology and capitalization of structural and non-structural protein domains are consistent with the main text. No additional changes were required.

General Scholarship & Significance

This is a high-quality, methodologically innovative manuscript that addresses a massive operational bottleneck in tropical medicine: the extensive serological cross-reactivity that compromises traditional immunoassay surveillance for co-circulating arboviruses. The execution is outstanding. By integrating innovative protein architecture (SNAP-tag molecular orientation) with a modern two-step machine learning workflow (Boruta-tuned Random Forests), the authors push serosurveillance beyond binary qualitative classification into quantitative functional assessments. Key Strengths
Epidemiological Diversity: Validation across four distinct, well-characterized global settings strengthens the biological context of the assay's performance.

Algorithmic Rigor: The machine learning approach is handled appropriately, transparently demonstrating where binding titers function well as a surrogate for neutralisation (CHIKV) and precisely where they fail to do so (DENV).

Methodological Transparency: Full protocol details for automated plate washers and KingFisher magnetic processors ensure excellent reproducibility.

We sincerely thank the reviewer for their highly positive assessment of our manuscript and for recognizing the methodological innovation, rigor, and translational relevance of our work. We greatly appreciate the reviewer’s acknowledgment of the strengths of our study, including the diversity of epidemiological settings, the integration of SNAP-tag antigen engineering with machine learning approaches, and the transparency of our methodological descriptions.

Minor Weaknesses

The small size of the target serotype PCR-confirmed reference panels slightly limits the evaluation of individual DENV serotype kinetic trends. However, the authors explicitly frame this as a limitation, which is acceptable for a study of this scale.

We also thank the reviewer for recognizing the limitations associated with the size of the PCR-confirmed serotype-specific samples. We agree that these limitations restrict the interpretation of individual DENV serotype kinetic trends.

No dual publication or ethical concerns exist. The authors have effectively standardized their terminology and addressed all historical review comments. This manuscript will make an excellent addition to PLOS Neglected Tropical Diseases.

Finally, we appreciate the reviewer’s acknowledgment that previous comments have been addressed and that the manuscript has been improved in terms of terminology, clarity, and overall presentation. We are grateful for the reviewer’s positive evaluation of the significance and potential impact of this work.
