# Supplementary material for: A multiplex serological assay for quantification of arbovirus antibody kinetics and neutralizing antibodies
Source: PLoS Negl Trop Dis. 2026 Sep 23;20(9):e0013704. doi: 10.1371/journal.pntd.0013704 (PMC13626472; doi:10.1371/journal.pntd.0013704)
Supplement: Peer review history

# Peer review: Decision Letter 0

**DOI:** 10.1371/journal.pntd.0013704.r001

**Authors:** Abdallah Samy

15 Jan 2026

A Multiplex Serological Assay for Quantification of Arbovirus Antibody Kinetics and Neutralizing Antibodies

PLOS Neglected Tropical Diseases

Dear Dr. White,

Thank you for submitting your manuscript to PLOS Neglected Tropical Diseases. After careful consideration, we feel that it has merit but does not fully meet PLOS Neglected Tropical Diseases's publication criteria as it currently stands. Therefore, we invite you to submit a revised version of the manuscript that addresses the points raised during the review process.

Please submit your revised manuscript within by Mar 16 2026 11:59PM. If you will need more time than this to complete your revisions, please reply to this message or contact the journal office at plosntds@plos.org. When you're ready to submit your revision, log on to https://www.editorialmanager.com/pntd/ and select the 'Submissions Needing Revision' folder to locate your manuscript file.

Please include the following items when submitting your revised manuscript:

* A letter that responds to each point raised by the editor and reviewer(s). You should upload this letter as a separate file labeled 'Response to Reviewers'. This file does not need to include responses to any formatting updates and technical items listed in the 'Journal Requirements' section below.

* A marked-up copy of your manuscript that highlights changes made to the original version. You should upload this as a separate file labeled 'Revised Manuscript with Track Changes'.

* An unmarked version of your revised paper without tracked changes. You should upload this as a separate file labeled 'Manuscript'.

If you would like to make changes to your financial disclosure, competing interests statement, or data availability statement, please make these updates within the submission form at the time of resubmission. Guidelines for resubmitting your figure files are available below the reviewer comments at the end of this letter.

We look forward to receiving your revised manuscript.

Kind regards,

Abdallah Samy

Section Editor

PLOS Neglected Tropical Diseases

Shaden Kamhawi

co-Editor-in-Chief

PLOS Neglected Tropical Diseases

orcid.org/0000-0003-4304-636XX

Paul Brindley

co-Editor-in-Chief

PLOS Neglected Tropical Diseases

orcid.org/0000-0003-1765-0002

**Additional Editor Comments:**

Please give careful consideration to all reviewer comments and ensure that each concern is thoroughly addressed prior to the submission of a revised version of your manuscript.

**Journal Requirements:**

If the reviewer comments include a recommendation to cite specific previously published works, please review and evaluate these publications to determine whether they are relevant and should be cited. There is no requirement to cite these works unless the editor has indicated otherwise.

1) Please ensure that the CRediT author contributions listed for every co-author are completed accurately and in full.

At this stage, the following Authors/Authors require contributions: Laura Garcia, Victor Yman, Marie-Fabrice Gasasira, Françoise Donnadieu, Lucie Cappuccio, Damien Hoinard, Philippe Desprès, Jean-Claude Manuguerra, In-Kyu Yoon, Taweewan Hunsawong, Maria Theresa P Alera, Emily S. Gurley, Nikos Vasilakis, Scott C. Weaver, Ziaur Rahman, Ilaria Dorigatti, Chris Drakeley, Inès Vigan-Womas, Makhtar Niang, Henrik Salje, Jessica Vanhomwegen, and Michael White. Please ensure that the full contributions of each author are acknowledged in the "Add/Edit/Remove Authors" section of our submission form.

The list of CRediT author contributions may be found here: https://journals.plos.org/plosntds/s/authorship#loc-author-contributions

2) Please provide an Author Summary. This should appear in your manuscript between the Abstract (if applicable) and the Introduction, and should be 150-200 words long. The aim should be to make your findings accessible to a wide audience that includes both scientists and non-scientists. Sample summaries can be found on our website under Submission Guidelines:

https://journals.plos.org/plosntds/s/submission-guidelines#loc-parts-of-a-submission

3) We do not publish any copyright or trademark symbols that usually accompany proprietary names, eg ©,  ®, or TM  (e.g. next to drug or reagent names). Therefore please remove all instances of trademark/copyright symbols throughout the text, including:

- ® on page: 9

- TM on page: 9.

4) Thank you for including an Ethics Statement for your study. Please include:

i) A statement that formal consent was obtained (must state whether verbal/written) OR the reason consent was not obtained (e.g. anonymity). NOTE: If child participants, the statement must declare that formal consent was obtained from the parent/guardian.].

5) Please upload all main figures as separate Figure files in .tif or .eps format. For more information about how to convert and format your figure files please see our guidelines:

https://journals.plos.org/plosntds/s/figures

6) We have noticed that you have uploaded Supporting Information files, but you have not included a list of legends. Please add a full list of legends for your Supporting Information files after the references list.

7) Please amend your detailed Financial Disclosure statement. This is published with the article. It must therefore be completed in full sentences and contain the exact wording you wish to be published.

1) State what role the funders took in the study. If the funders had no role in your study, please state: "The funders had no role in study design, data collection and analysis, decision to publish, or preparation of the manuscript."

2) If any authors received a salary from any of your funders, please state which authors and which funders..

If you did not receive any funding for this study, please simply state: u201cThe authors received no specific funding for this work.u201d

8)  Please ensure that the funders and grant numbers match between the Financial Disclosure field and the Funding Information tab in your submission form. Note that the funders must be provided in the same order in both places as well.

**Reviewers' Comments:**

Reviewer's Responses to Questions

**Key Review Criteria Required for Acceptance?**

As you describe the new analyses required for acceptance, please consider the following:

**Methods**

-Are the objectives of the study clearly articulated with a clear testable hypothesis stated?

-Is the study design appropriate to address the stated objectives?

-Is the population clearly described and appropriate for the hypothesis being tested?

-Is the sample size sufficient to ensure adequate power to address the hypothesis being tested?

-Were correct statistical analysis used to support conclusions?

-Are there concerns about ethical or regulatory requirements being met?

Reviewer #1: In the manuscript “A Multiplex Serological Assay for Quantification of Arbovirus Antibody Kinetics and Neutralizing Antibodies”, the authors developed and evaluated a multiplex bead-based immunoassay to assess Ab responses against a panel of arboviruses.

My main concern is that there are many similar assays described in the literature and many bead-based serology tests available in the market. Thus, what additional features does the described method add to the state of the art? In the introduction, for instance, the authors acknowledge one such study (reference 14), but the number of similar studies and commercial kits is much greater!

At first, an obvious differential is the large panel of antigens evaluated in the study, including native or recombinant proteins from orthoflaviviruses, CHKV, Mayaro virus, WNV, and a few others. The antigens were largely based on either surface proteins or soluble non-structural ones (like flaviviruses’ NS1). Some of the antigens were tagged with an O6-alkylguanine-DNA alkyltransferase enzyme domain (SNAP) before linking to the beads.

Reviewer #2: The study objectives are clearly stated, and the overall goal—to develop and evaluate a multiplex arbovirus serological assay—is well articulated. However, the manuscript would benefit from a more explicit statement of the central hypothesis regarding antigen-specific discrimination of infection history. The study design is appropriate, incorporating both cross-sectional and longitudinal samples and using PCR and PRNT confirmation where possible. The populations from different geographic regions are adequately described and relevant to the intended application of the assay. While overall sample numbers are large, subsets with PCR-confirmed infections remain limited, particularly for dengue serotypes, reducing power for antigen-level performance assessment and machine-learning validation. Statistical methods used, including correlation analyses and Random Forest models, are appropriate, though the description of validation (e.g., OOB error, mitigation of overfitting) should be clearer. No major ethical concerns are evident, but additional detail on IRB approvals and consent procedures across sites would strengthen the methods section.

**********

**Results**

-Does the analysis presented match the analysis plan?

-Are the results clearly and completely presented?

-Are the figures (Tables, Images) of sufficient quality for clarity?

Reviewer #1: Despite the large panel of antigens from various arboviruses, results were not different from those seen in many other papers, showing that surface antigens are the most appropriate for arboviruses’ serosurvey. NS1 responses in orthoflaviviruses, such as dengue, are also present and useful, as expected.

The authors acknowledge the possibility of intense serological cross-reactivity between related viruses (within Flaviviruses, or within alphaviruses, for instance); therefore, the presented bead-based assay appears not to have added any considerable specificity gain to the field.

Additionally, the manuscript suffers from a certain “identity crisis”, if I may. At the same time that it introduces the Multiplex Serological Assay as a new tool, it also seems to focus on partial serological studies using sera samples from various locations. At the end, it is my impression that neither objective is accomplished in enough depth.

Finally, the study describes a “two-step random forest modelling approach to assess whether the multiplex serological data could be used to predict PRNT titers for DENV”. Again, although results are fairly good in relation to CHKV, when it comes to dengue, however, the correlation between total IgG and NAbs becomes less precise.

Reviewer #2: The analyses presented align well with the methods and objectives. Results are comprehensive, demonstrating antigen reactivity patterns, cross-reactivity within flaviviruses, and strong CHIKV assay performance. Presentation of cross-reactivity is clear but would benefit from more quantitative summaries such as antigen correlation matrices or ROC metrics. The findings on SNAP-tagged antigens are promising, though more explicit comparison of performance versus conventional antigens would improve clarity. Figures generally convey the data but are often dense, making interpretation challenging; simplified panel structures or summary graphics would enhance readability. Overall, the data are presented clearly and support the minor conclusions.

**********

**Conclusions**

-Are the conclusions supported by the data presented?

-Are the limitations of analysis clearly described?

-Do the authors discuss how these data can be helpful to advance our understanding of the topic under study?

-Is public health relevance addressed?

Reviewer #1: Despite the obvious amount of work and parameters included in the multiplex bead-based immunoassay, the obtained results have not added much to the current state-of-the-art related to similar tests. Nor has it substantially contributed to serosurvey studies using the available sera banks. It is my opinion that the authors should focus on a better characterization of the developed kit and compare its performance to either published or commercially available kits. As an example, a quick search in the literature retrieved many important similar studies that were not mentioned in the current manuscript (a few examples - not limited to these: https://doi.org/10.1128/CVI.05537-11 ; https://doi.org/10.3390/tropicalmed10040092 ; https://doi.org/10.1038/s41598-025-96769-y ; doi:10.3791/69259 )

Reviewer #2: The conclusions are broadly supported by the data. The manuscript appropriately identifies strengths such as the robust performance of CHIKV antigens and limitations posed by extensive flavivirus cross-reactivity. However, claims regarding serotype distinction and PRNT prediction should be moderated given small sample sizes. The discussion includes relevant limitations but could more clearly describe how sample constraints and potential overfitting affect generalizability. Public health relevance is addressed, though additional context regarding assay feasibility, cost, and deployment would strengthen translational impact. Overall, the conclusions reflect the value of the assay while acknowledging key challenges.

**********

**Editorial and Data Presentation Modifications?**

Use this section for editorial suggestions as well as relatively minor modifications of existing data that would enhance clarity. If the only modifications needed are minor and/or editorial, you may wish to recommend “Minor Revision” or “Accept”.

Reviewer #1: N/A

Reviewer #2: Several editorial refinements would improve clarity. Figures should be simplified, with key results emphasized and complex panels reorganized. Consistency in virus nomenclature and antigen naming is needed. Minor typographical errors should be corrected. Additional quantitative summaries—correlation heatmaps, ROC curves, and more explicit PRNT-serology comparisons—would enhance interpretability without requiring new experiments. Clarifying antigen engineering benefits and providing a brief diagram of sample flow would strengthen the manuscript. These are minor revisions and focus primarily on presentation and clarity rather than new data generation.

**********

**Summary and General Comments**

Use this section to provide overall comments, discuss strengths/weaknesses of the study, novelty, significance, general execution and scholarship. You may also include additional comments for the author, including concerns about dual publication, research ethics, or publication ethics. If requesting major revision, please articulate the new experiments that are needed.

Reviewer #1: Despite the obvious amount of work and parameters included in the multiplex bead-based immunoassay, the obtained results have not added much to the current state-of-the-art related to similar tests. Nor has it substantially contributed to serosurvey studies using the available sera banks. It is my opinion that the authors should focus on a better characterization of the developed kit and compare its performance to either published or commercially available kits. As an example, a quick search in the literature retrieved many important similar studies that were not mentioned in the current manuscript (a few examples - not limited to these: https://doi.org/10.1128/CVI.05537-11 ; https://doi.org/10.3390/tropicalmed10040092 ; https://doi.org/10.1038/s41598-025-96769-y ; doi:10.3791/69259 )

Reviewer #2: This is a strong, well-executed study with substantial relevance for arbovirus surveillance. The multiplex assay, broad antigen panel, and integration of PRNT validation and machine-learning prediction represent meaningful contributions to the field. Strengths include diverse cohorts, longitudinal data, and innovative antigen engineering. Weaknesses include limited PCR-confirmed samples for certain viruses, descriptive rather than quantitative evaluation of cross-reactivity, and insufficient model validation details. With revisions focused on data presentation, clearer statistical justification, and improved contextual framing, the manuscript has high potential for publication. Recommended: Minor Revision.

**********

PLOS authors have the option to publish the peer review history of their article (what does this mean?). If published, this will include your full peer review and any attached files.

If you choose “no”, your identity will remain anonymous but your review may still be made public.

**Do you want your identity to be public for this peer review?**  For information about this choice, including consent withdrawal, please see our Privacy Policy.

Reviewer #1: No

Reviewer #2: No

**Figure resubmission:**

While revising your submission, we strongly recommend that you use PLOS’s NAAS tool (https://ngplosjournals.pagemajik.ai/artanalysis) to test your figure files. NAAS can convert your figure files to the TIFF file type and meet basic requirements (such as print size, resolution), or provide you with a report on issues that do not meet our requirements and that NAAS cannot fix.

**Reproducibility:**

To enhance the reproducibility of your results, we recommend that authors of applicable studies deposit laboratory protocols in protocols.io, where a protocol can be assigned its own identifier (DOI) such that it can be cited independently in the future. Additionally, PLOS ONE offers an option to publish peer-reviewed clinical study protocols. Read more information on sharing protocols at https://plos.org/protocols?utm_medium=editorial-email&utm_source=authorletters&utm_campaign=protocols

# Author comment: Author response to Decision Letter 1

**DOI:** 10.1371/journal.pntd.0013704.r002

12 Mar 2026

Attachment Submitted filename: Garcia_PNTD-D-25-01903_Response_to_Editorial_Requests.docx

# Peer review: Decision Letter 1

**DOI:** 10.1371/journal.pntd.0013704.r003

**Authors:** Abdallah Samy

12 May 2026

Response to Reviewers Revised Manuscript with Track Changes Manuscript

Shaden Kamhawi

co-Editor-in-Chief

PLOS Neglected Tropical Diseases

orcid.org/0000-0003-4304-636XX

Paul Brindley

co-Editor-in-Chief

PLOS Neglected Tropical Diseases

orcid.org/0000-0003-1765-0002

**Additional Editor Comments:** **Journal Requirements:**

1) We have noticed that you have uploaded Supporting Information files, but you have not included a complete list of legends. Please add a full list of legends for your Supporting Information files after the references list.

**Reviewers' comments:**

**Key Review Criteria Required for Acceptance?**

As you describe the new analyses required for acceptance, please consider the following:

**Methods**

-Are the objectives of the study clearly articulated with a clear testable hypothesis stated?

-Is the study design appropriate to address the stated objectives?

-Is the population clearly described and appropriate for the hypothesis being tested?

-Is the sample size sufficient to ensure adequate power to address the hypothesis being tested?

-Were correct statistical analysis used to support conclusions?

-Are there concerns about ethical or regulatory requirements being met?

Reviewer #1: No further observation regarding methodology.

Reviewer #2: Objectives and Hypothesis: The objectives are clearly articulated: to develop and validate a high-throughput multiplex assay and to assess its ability to predict neutralizing antibody titers using machine learning. The authors effectively state the aim of exploring antibody kinetics following PCR-confirmed infections.

Study Design and Population: The design is appropriate, utilizing 647 samples from four countries (Senegal, France, Philippines, and Bangladesh) to represent both endemic and non-endemic regions.

Sample Size: While the total sample size is robust for general distribution analysis, the authors and reviewers acknowledge that the subset of PCR-confirmed infections—particularly for specific dengue serotypes—is limited.

Statistical Analysis: The two-step Random Forest (RF) modeling approach, incorporating Boruta feature selection and out-of-bag (OOB) internal validation, is statistically sound for this type of high-dimensional serological data.

Ethics: Regulatory requirements are well-documented, including specific IRB protocol numbers and a clear statement that written informed consent was obtained from all participants or guardians.

**********

**Results**

-Does the analysis presented match the analysis plan?

-Are the results clearly and completely presented?

-Are the figures (Tables, Images) of sufficient quality for clarity?

Reviewer #1: Please, see below.

Reviewer #2: Presentation: The results are presented logically, moving from general IgG distributions to longitudinal kinetics and finally to predictive modeling.

Key Findings: * The assay identified CHIKV E2 and VLP, and DENV1 EDIII, as strong markers for serosurveillance.

Predictive modeling for CHIKV neutralization was highly successful ($R^2=0.71$), whereas DENV1 prediction was significantly weaker ($R^2=0.42$), highlighting the complexity of orthoflavivirus cross-reactivity.

Data Quality: Figures (1–6) are of high quality and provide clear visualizations of antibody distributions and model performance. The inclusion of a commercial comparison with the Tetracore kit strengthens the validation.

**********

**Conclusions**

-Are the conclusions supported by the data presented?

-Are the limitations of analysis clearly described?

-Do the authors discuss how these data can be helpful to advance our understanding of the topic under study?

-Is public health relevance addressed?

Reviewer #1: Please, see below.

Reviewer #2: Data Support: The conclusions are directly supported by the data, particularly the emphasized difficulty of using binding antibodies to predict neutralization for orthoflaviviruses.

Limitations: The authors candidly discuss limitations, including the small number of PCR-confirmed reference samples and the inability to distinguish between natural yellow fever infection and vaccination.

Significance: The study highlights the utility of multiplexing for immunosurveillance in regions where multiple arboviruses co-circulate, offering a scalable alternative to labor-intensive PRNTs.

**********

**Editorial and Data Presentation Modifications?**

Use this section for editorial suggestions as well as relatively minor modifications of existing data that would enhance clarity. If the only modifications needed are minor and/or editorial, you may wish to recommend “Minor Revision” or “Accept”.

Reviewer #1: Please, see below

Reviewer #2: The authors have addressed previous reviewer concerns by adding a quantitative correlation heatmap (Supplementary Figure S2) and clarifying the advantages of SNAP-tagged antigens.

Suggestion: Ensure consistent nomenclature for all antigens across text and figures (e.g., standardizing the use of "EDIII" vs "domain III") to maximize readability.

**********

**Summary and General Comments**

Use this section to provide overall comments, discuss strengths/weaknesses of the study, novelty, significance, general execution and scholarship. You may also include additional comments for the author, including concerns about dual publication, research ethics, or publication ethics. If requesting major revision, please articulate the new experiments that are needed.

Reviewer #1: The authors have satisfactorily responded to almost all raised concerns. Nonetheless, there are still some remaining details that need addressing.

For instance, in their rebuttal letter, the authors stated that they “have added more references in the introduction (line 114)” in order to acknowledge the current state of the art concerning similar diagnostic approaches. However, when I look at line 114 (as per the indication), I see no added references.

Another concern was to make it clear that this is not a serosurvey study, but a methodological work using epidemiologically relevant samples to validate the tool. In this respect, author stated that they “have revised the manuscript to more explicitly emphasize this methodological focus and to avoid interpretation as a serosurvey (lines xx in the introduction). What are lines “xx”? A marked version of the revised manuscript is quite important to show reviewers what was modified in relation to the original submission.

It was also pointed out in the revision that the correlation between total IgG and NAbs is way less precise for DENV than it is for CHIKV. The authors agreed with that, but apparently have not made any additions to the paper in order to indicate such a limitation. That should be added.

Reviewer #2: This study represents a significant methodological contribution to arbovirus research. The primary strength lies in the integration of innovative antigen engineering (SNAP-tagging) with machine learning to interpret complex serological profiles.

While the predictive power for dengue neutralization remains a challenge—reflecting broader biological hurdles in flavivirus immunology—the assay’s high-throughput nature and small sample volume requirement make it a valuable tool for global health surveillance. The authors' revisions have successfully addressed prior criticisms regarding comparative performance and statistical clarity.

**********

PLOS authors have the option to publish the peer review history of their article (what does this mean?). If published, this will include your full peer review and any attached files.

If you choose “no”, your identity will remain anonymous but your review may still be made public.

**Do you want your identity to be public for this peer review?**  For information about this choice, including consent withdrawal, please see our Privacy Policy.

Reviewer #1: No

Reviewer #2: No

**Figure resubmission:**

**Reproducibility:**

To enhance the reproducibility of your results, we recommend that authors of applicable studies deposit laboratory protocols in protocols.io, where a protocol can be assigned its own identifier (DOI) such that it can be cited independently in the future. Additionally, PLOS ONE offers an option to publish peer-reviewed clinical study protocols. Read more information on sharing protocols at https://plos.org/protocols?utm_medium=editorial-email&utm_source=authorletters&utm_campaign=protocols

# Author comment: Author response to Decision Letter 2

**DOI:** 10.1371/journal.pntd.0013704.r004

29 May 2026

Attachment Submitted filename: Garcia_PNTD-D-25-01903_Response_to_Editorial_Points_29May.docx

# Peer review: Decision Letter 2

**DOI:** 10.1371/journal.pntd.0013704.r005

**Authors:** Abdallah Samy

27 Jul 2026

Response to Reviewers Revised Manuscript with Track Changes Manuscript

Shaden Kamhawi

co-Editor-in-Chief

PLOS Neglected Tropical Diseases

orcid.org/0000-0003-4304-636XX

Paul Brindley

co-Editor-in-Chief

PLOS Neglected Tropical Diseases

orcid.org/0000-0003-1765-0002

**Additional Editor Comments (if provided):** **Journal Requirements:**

**Reviewers' comments:**

**Key Review Criteria Required for Acceptance?**

As you describe the new analyses required for acceptance, please consider the following:

**Methods**

-Are the objectives of the study clearly articulated with a clear testable hypothesis stated?

-Is the study design appropriate to address the stated objectives?

-Is the population clearly described and appropriate for the hypothesis being tested?

-Is the sample size sufficient to ensure adequate power to address the hypothesis being tested?

-Were correct statistical analysis used to support conclusions?

-Are there concerns about ethical or regulatory requirements being met?

Reviewer #2: the objectives are clearly articulated. The authors explicitly state their goal: to develop, automate, and validate a high-throughput multiplex bead-based immunoassay to measure IgG, IgA, and IgM responses against a wide panel of 44 arbovirus antigens, and to explore antibody kinetics and predict plaque reduction neutralization test (PRNT) titers using machine learning. While framed more as a methodological validation than a rigid hypothesis-driven mechanistic paper, it clearly tests whether multiplex binding profiles can serve as a functional surrogate for virus-neutralizing activity. the study design is highly appropriate. It leverages a diverse panel of 647 samples stratified across four distinct geographic and epidemiologic regions: endemic settings (Senegal, Bangladesh, the Philippines) and a non-endemic control setting (France). It also cleverly incorporates both longitudinal cohorts with RT-PCR-confirmed infections to map kinetic boosts over time and cross-sectional panels with paired gold-standard neutralization data. the population cohorts are explicitly defined in the text and neatly organized in Table 1, providing details on expected exposure, sample size, median age, and sex distribution. Utilizing unexposed individuals from France alongside populations frequently exposed to co-circulating arboviruses provides the ideal physiological contrast needed to evaluate assay specificity and background cross-reactivity. The overall sample size ($n = 647$) is robust for mapping general antibody distributions and training the machine learning models. However, as the authors transparently acknowledge in the limitations, the subset of individuals with longitudinal, RT-PCR-confirmed infections is modest (e.g., 12 for CHIKV, 10 for DENV1, and single digits for other DENV serotypes). While sufficient for an exploratory descriptive study of kinetic trends, it limits broader statistical generalization of serotype-specific kinetics. The two-step Random Forest framework—separating classification (PRNT positive vs. negative) from regression (predicting log-titers)—coupled with Boruta feature selection is a statistically sound approach for handling high-dimensional multiplex array inputs. The use of out-of-bag (OOB) predictions provides a reliable form of internal cross-validation. there are no concerns. Institutional Review Board (IRB) approval details and explicit protocol registry numbers are documented for all four study sites. In response to prior editorial adjustments, the text now explicitly declares that written formal informed consent was obtained from all participants or their legal guardians.

**********

**Results**

-Does the analysis presented match the analysis plan?

-Are the results clearly and completely presented?

-Are the figures (Tables, Images) of sufficient quality for clarity?

Reviewer #2: the presented results track the structured objectives perfectly. The sequence flows logically from defining distribution profiles across countries (Figures 1 and 2), exploring longitudinal antibody boosts post-infection (Figures 3 and 4), to executing the machine learning neutralization modeling (Figures 5 and 6). the data is detailed thoroughly. The manuscript beautifully highlights a crucial biological contrast: binding antibodies translate to an excellent surrogate model for predicting functional neutralization in alphaviruses (CHIKV R2 = 0.71) but perform poorly for orthoflaviviruses (DENV1 R2 = 0.42) due to intricate cross-reactive profiles and immunodominance issues. The figures are exceptionally well-crafted, clear, and informative. The multi-panel alignment comparing actual vs. predicted titers alongside Boruta variable importance plots makes complex algorithmic outputs easily digestible at a glance. The authors have successfully corrected their formatting in accordance with journal specifications.

**********

**Conclusions**

-Are the conclusions supported by the data presented?

-Are the limitations of analysis clearly described?

-Do the authors discuss how these data can be helpful to advance our understanding of the topic under study?

-Is public health relevance addressed?

Reviewer #2: the conclusions are well-supported. The data directly confirms that while a multiplex approach can vastly optimize high-throughput epidemiological surveillance, using total IgG binding metrics to infer functional immunity or specific neutralisation thresholds remains highly restricted for flaviviruses. The authors are candid about their biological and physical constraints. They discuss the small sample counts for PCR-confirmed reference cohorts, the lack of broader commercial reference standards, and the intrinsic inability of this platform to reliably distinguish between past natural yellow fever exposure and vaccination responses. The discussion carefully outlines how implementing site-specifically oriented, SNAP-tagged engineered envelope domains (EDIII and E2) can bypass the typical architectural pitfalls of random amine coupling. This advances serological method development by significantly lowering background noise and nonspecific cross-reactivity. Extensively. The authors emphasize the scalability, low sample volume demands, and cost-efficiency of this multiplex platform. They cleanly link the tool to its real-world implementation: mapping multi-pathogen transmission landscapes and shaping vaccine deployment strategies in areas plagued by co-circulating arboviruses.

**********

**Editorial and Data Presentation Modifications?**

Use this section for editorial suggestions as well as relatively minor modifications of existing data that would enhance clarity. If the only modifications needed are minor and/or editorial, you may wish to recommend “Minor Revision” or “Accept”.

Reviewer #2: The manuscript is in excellent shape, reflecting rigorous responses to prior evaluation rounds. Only minor, optional formatting enhancements are suggested to polish readability:

Aesthetics in Figures 3 and 4: In Figure 3A and Figure 4A, some of the light gray line traces representing "other alphaviruses" or "other orthoflaviviruses" crowd the background of the individual facets. While acceptable, making these gray lines slightly more translucent would let the primary colored virus traces pop even more effectively.

Abbreviation Continuity: Check that all structural domains are consistently abbreviated following the authors' standardization exercise. Ensure that "structural polyprotein" (SP) or "non-structural protein" (NSP) are fully capitalized across all supplementary figure subtitles to match the main text layout.

**********

**Summary and General Comments**

Use this section to provide overall comments, discuss strengths/weaknesses of the study, novelty, significance, general execution and scholarship. You may also include additional comments for the author, including concerns about dual publication, research ethics, or publication ethics. If requesting major revision, please articulate the new experiments that are needed.

Reviewer #2: General Scholarship & Significance

This is a high-quality, methodologically innovative manuscript that addresses a massive operational bottleneck in tropical medicine: the extensive serological cross-reactivity that compromises traditional immunoassay surveillance for co-circulating arboviruses. The execution is outstanding. By integrating innovative protein architecture (SNAP-tag molecular orientation) with a modern two-step machine learning workflow (Boruta-tuned Random Forests), the authors push serosurveillance beyond binary qualitative classification into quantitative functional assessments. Key Strengths

Epidemiological Diversity: Validation across four distinct, well-characterized global settings strengthens the biological context of the assay's performance.

Algorithmic Rigor: The machine learning approach is handled appropriately, transparently demonstrating where binding titers function well as a surrogate for neutralisation (CHIKV) and precisely where they fail to do so (DENV).

Methodological Transparency: Full protocol details for automated plate washers and KingFisher magnetic processors ensure excellent reproducibility.

Minor Weaknesses

The small size of the target serotype PCR-confirmed reference panels slightly limits the evaluation of individual DENV serotype kinetic trends. However, the authors explicitly frame this as a limitation, which is acceptable for a study of this scale.

No dual publication or ethical concerns exist. The authors have effectively standardized their terminology and addressed all historical review comments. This manuscript will make an excellent addition to PLOS Neglected Tropical Diseases.

**********

PLOS authors have the option to publish the peer review history of their article (what does this mean?). If published, this will include your full peer review and any attached files.

If you choose “no”, your identity will remain anonymous but your review may still be made public.

**Do you want your identity to be public for this peer review?**  For information about this choice, including consent withdrawal, please see our Privacy Policy.

Reviewer #2: No

**Figure resubmission:**

**Reproducibility:**

To enhance the reproducibility of your results, we recommend that authors of applicable studies deposit laboratory protocols in protocols.io, where a protocol can be assigned its own identifier (DOI) such that it can be cited independently in the future. Additionally, PLOS ONE offers an option to publish peer-reviewed clinical study protocols. Read more information on sharing protocols at https://plos.org/protocols?utm_medium=editorial-email&utm_source=authorletters&utm_campaign=protocols

# Author comment: Author response to Decision Letter 3

**DOI:** 10.1371/journal.pntd.0013704.r006

6 Aug 2026

Attachment Submitted filename: responses_to_reviewers.docx

# Editor report: Decision Letter 3

**DOI:** 10.1371/journal.pntd.0013704.r007

**Authors:** Abdallah Samy

4 Sep 2026

Dear Dr. White,

We are pleased to inform you that your manuscript 'A Multiplex Serological Assay for Quantification of Arbovirus Antibody Kinetics and Neutralizing Antibodies' has been provisionally accepted for publication in PLOS Neglected Tropical Diseases.

Before your manuscript can be formally accepted you will need to complete some formatting changes, which you will receive in a follow up email. A member of our team will be in touch with a set of requests.

Please note that your manuscript will not be scheduled for publication until you have made the required changes, so a swift response is appreciated.

IMPORTANT: The editorial review process is now complete. PLOS will only permit corrections to spelling, formatting or significant scientific errors from this point onwards. Requests for major changes, or any which affect the scientific understanding of your work, will cause delays to the publication date of your manuscript.

Should you, your institution's press office or the journal office choose to press release your paper, you will automatically be opted out of early publication. We ask that you notify us now if you or your institution is planning to press release the article. All press must be co-ordinated with PLOS.

Thank you again for supporting Open Access publishing; we are looking forward to publishing your work in PLOS Neglected Tropical Diseases.

Best regards,

Abdallah M. Samy, PhD

Section Editor

PLOS Neglected Tropical Diseases

Abdallah Samy

Section Editor

PLOS Neglected Tropical Diseases

Shaden Kamhawi

co-Editor-in-Chief

PLOS Neglected Tropical Diseases

orcid.org/0000-0003-4304-636XX

Paul Brindley

co-Editor-in-Chief

PLOS Neglected Tropical Diseases

orcid.org/0000-0003-1765-0002

***********************************************************

# Editor report: Acceptance letter

**DOI:** 10.1371/journal.pntd.0013704.r008

**Authors:** Abdallah Samy

Dear Dr. White,

We are delighted to inform you that your manuscript, "A Multiplex Serological Assay for Quantification of Arbovirus Antibody Kinetics and Neutralizing Antibodies," has been formally accepted for publication in PLOS Neglected Tropical Diseases.

We have now passed your article onto the PLOS Production Department who will complete the rest of the publication process. All authors will receive a confirmation email upon publication.

The corresponding author will soon be receiving a typeset proof for review, to ensure errors have not been introduced during production. Please review the PDF proof of your manuscript carefully, as this is the last chance to correct any scientific or type-setting errors. Please note that major changes, or those which affect the scientific understanding of the work, will likely cause delays to the publication date of your manuscript. Note: Proofs for Front Matter articles (Editorial, Viewpoint, Symposium, Review, etc...) are generated on a different schedule and may not be made available as quickly.

Soon after your final files are uploaded, the early version of your manuscript will be published online unless you opted out of this process. The date of the early version will be your article's publication date. The final article will be published to the same URL, and all versions of the paper will be accessible to readers.

For Research Articles, you will receive an invoice from PLOS for your publication fee after your manuscript has reached the completed accept phase. If you receive an email requesting payment before acceptance or for any other service, this may be a phishing scheme. Learn how to identify phishing emails and protect your accounts at https://explore.plos.org/phishing.

Thank you again for supporting open-access publishing; we are looking forward to publishing your work in PLOS Neglected Tropical Diseases.

Best regards,

Shaden Kamhawi

co-Editor-in-Chief

PLOS Neglected Tropical Diseases

Paul Brindley

co-Editor-in-Chief

PLOS Neglected Tropical Diseases
